# Metabolic Syndrome and Obesity‐related cancer Risk and Survival: An Umbrella Review of Systematic Reviews With Meta‐analysis of Observational Studies

**DOI:** 10.1111/obr.70073

**Published:** 2026-01-08

**Authors:** Maci Winn, Prasoona Karra, Ryzen Benson, Svenja Pauleck, Nathorn Chaiyakunapruk, Win Khaing, Sajesh K. Veettil, Mary M. McFarland, Tallie Casucci, Yizhe Xu, Siwen Hu‐Lieskovan, Michelle Litchman, Mary Playdon, Sheetal Hardikar

**Affiliations:** ^1^ Department of Population Health Sciences University of Utah Salt Lake City Utah USA; ^2^ Huntsman Cancer Institute Salt Lake City Utah USA; ^3^ Department of Nutrition and Integrative Physiology University of Utah Salt Lake City Utah USA; ^4^ Department of Epidemiology Geisel School of Medicine at Dartmouth College Lebanon New Hampshire USA; ^5^ University of California San Francisco Bakar Computational Health Sciences Institute San Francisco California USA; ^6^ Department of Radiation Oncology University of California San Francisco San Francisco California USA; ^7^ College of Pharmacy University of Utah Salt Lake City Utah USA; ^8^ Department of Pharmacy Practice International Medical University Kuala Lumpur Malaysia; ^9^ University of Utah Spencer S. Eccles Health Sciences Library Salt Lake City Utah USA; ^10^ University of Utah J. Willard Marriott Library Salt Lake City Utah USA; ^11^ University of Utah Department of Internal Medicine, Division of Epidemiology Salt Lake City Utah USA; ^12^ College of Nursing University of Utah Salt Lake City Utah USA

**Keywords:** cancer risk, cancer survival, epidemiology, metabolic syndrome, obesity‐related cancer, systematic review, umbrella review

## Abstract

**Introduction:**

Metabolic syndrome (MetS) may be associated with obesity‐related cancer (ORC) owing to shared risk factors like physical inactivity, insulin resistance, gut microbiome dysfunction, and inflammation. We conducted an umbrella review of systematic reviews with meta‐analysis to synthesize the evidence on the association between MetS and ORC risk and survival.

**Methods:**

Searches in five databases (Medline, Embase, CINAHL, Cochrane Library, and Scopus) retrieved 2524 systematic reviews with meta‐analyses (SRMAs), which underwent title and abstract screening (2524), full‐text review (41), and data extraction for included SRMAs (21). Summary effects and 95% confidence intervals were re‐estimated using random‐effects models. Methodological quality, certainty of evidence, and publication bias were assessed using the AMSTAR 2, modified Ioannidis criteria, and Egger's test, respectively.

**Results:**

A total of 25 associations between MetS and ORC risk and five between MetS and survival were evaluated. Overall, 10 associations evaluating MetS and ORC risk were highly suggestive (four) or suggestive (six), while the rest were classified as weak (seven) or nonsignificant (eight). One association was suggestive for MetS and ORC survival, while the rest were classified as weak (three) or nonsignificant (one). The Egger's and excess significance tests were significant for 8(32%) associations between MetS and ORC risk and 3(60%) associations between MetS and ORC survival.

**Conclusion:**

This umbrella review suggests metabolic syndrome increases the risk of several obesity‐related cancers and worsens colorectal cancer survival. Despite study variability, consistent associations across diverse populations highlight the urgency of prevention and management strategies targeting metabolic dysfunction to reduce cancer burden.

**Summary**

In this umbrella review, highly suggestive and suggestive evidence supports associations between MetS and the risk and survival of several obesity‐related cancers. However, a better understanding of the relationship between metabolic syndrome and obesity‐related cancers is still needed to provide appropriate clinical care, design optimal interventions, and prevent subsequent increases in the risks of cancer, morbidity, and mortality.

## 1 INTRODUCTION

Metabolic syndrome (MetS), a group of risk factors, including hyperglycemia, dyslipidemia, hypertension, and central adiposity, is often used clinically to define metabolic health and cardiometabolic disease risk in adults [1]. MetS is driven by genetics and behavioral and environmental factors, including obesity, poor diet, physical inactivity, circadian cycle disruption, gastrointestinal dysbiosis, smoking, alcohol consumption, and stress [2, 3, 4, 5, 6, 7]. Up to one‐third of adults in developed countries have MetS, with rising rates due to aging populations [8]. MetS is also linked to cancer risk owing to shared risk factors and underlying etiological mechanisms [9, 10].

Obesity, a key component of MetS, increases the risk of at least thirteen cancer types called obesity‐related cancers (ORCs), including breast, colorectal, endometrial, ovarian, thyroid, renal‐cell, pancreatic, liver, gallbladder, multiple myeloma, meningioma, gastric cardia, and esophageal adenocarcinoma [11]. Despite prevention efforts, ORC incidence is rising, highlighting the public health impact of MetS on future cancer rates and survival [12]. Additionally, body mass index (BMI), the most common obesity metric, fails to identify up to a third of normal‐weight (BMI 18.5‐ < 25 kg/m^2^) individuals with metabolic dysfunction who may be at increased cancer risk [13, 14]. Thus, evaluating MetS as part of standard clinical care may aid in cancer risk stratification. Finally, although systematic reviews with meta‐analyses (SRMAs) have examined the link between MetS and ORC risk and survival, varying definitions of MetS and unequivocal evidence for specific cancers exist.

This umbrella review aimed to systematically identify relevant SRMAs of MetS and ORC risk and survival, synthesize their findings, and assess the strength and certainty of evidence to provide robust foundational evidence for the magnitude and credibility of the associations between MetS and ORC risk and survival.

## 2 Materials & Methods

This umbrella review was conducted using methods specified in the *Cochrane Handbook for Systematic Reviews of Interventions version 6.0* and in *Joanna Briggs Institute (JBI) Reviewer's Manual* while adhering to PRISMA reporting guidelines [15, 16, 17, 18, 19, 20]. The protocol was registered in PROSPERO (CRD42021230899) [21]. Protocol changes with justifications are in Appendix A.

### 2.1 Eligibility Criteria

The research question was developed using the Population, phenomena of Interest, Context, and Outcomes (PICo) structure [(P) Adults (≥ 18 years), (I) MetS, (C) Any geographic location or setting, and (O) ORC incidence or survival (overall or ORC‐specific)] [15]. ORCs were defined per the International Agency for Research on Cancer determination (including colorectal, postmenopausal breast, endometrial, ovarian, kidney, esophageal adenocarcinoma, gastric cardia, liver, gallbladder, pancreatic, meningioma, multiple myeloma, and thyroid cancers) [11].

### 2.2 Information Sources and Search Strategy

A search using Medline (Ovid) 1946–2023, Embase (Elsevier) 1974–2023, Cumulated Index to Nursing and Allied Health Literature (CINAHL) Complete (Ebscohost) 1937–2023, Cochrane Library (Wiley) 1898–2023, and Scopus (Elsevier) 1970–2023 databases was conducted using a combination of database‐specific subject headings and Keywords for the concepts of MetS and ORCs with filters for SRMAs of observational studies (Appendix B). Searches were conducted by a University of Utah librarian (T.C.) and peer‐reviewed by an information specialist (M.M.M.) according to PRESS guidelines [22]. The database search Results were updated on January 3, 2023, from the original searches conducted in February 2021. No publication date limits were applied. EndNote x20 (Clarivate) was used for citation management and article duplication removal, with Covidence (Veritas Health Innovation) providing a secondary means for removing duplicates.

### 2.3 Selection Process

Using Covidence, two reviewers (M.W. and P.K.) independently screened titles and abstracts and reviewed full texts. Discrepancies were resolved by a third reviewer (S.H. or M.P.). The reference lists from included reviews were reviewed (M.W.) to identify additional SRMAs.

### 2.4 Data Collection Process

Two investigators (M.W. and R.B.) independently extracted data from SRMAs using the JBI data extraction form customized to fit the research question [15]. Extraction items included descriptive characteristics, study design, database search information, risk of bias assessments, appraisal instrument and ratings, measures of heterogeneity, analysis methods, and findings. Disagreements were resolved by a third reviewer (S.H. or M.P.). All extracted data were verified in original research studies by at least two reviewers (M.W., S.P., P.K., R.B.) and corrected as necessary.

If sample size data were missing, we attempted to contact the original study authors and added the information received. For the quantitative analysis, when more than one SRMA was available for the same research question, we selected the SRMA with the highest number of studies. If more than one SRMA included the same number of studies, the newest review was selected.

### 2.5 Study Risk of bias Assessment

The methodological quality of each SRMA was assessed using the 16‐item AMSTAR 2 (A MeaSurement Tool to Assess systematic Reviews Version 2; M.W.) [23]. Seven domains considered critical for high‐quality studies were used to rate the overall confidence (high, moderate, low, or critically low‐quality) in each meta‐analysis [23] (Table S1). As a sensitivity analysis, we repeated the AMSTAR2 assessment, excluding two critical domains including 1) protocol registration, and 2) justification for excluded studies, to evaluate the impact of these criteria on overall quality ratings.

### 2.6 Effect Measures and Synthesis Methods

Summary effects and 95% confidence intervals (CIs) were re‐estimated using random‐effects models for MetS and ORC risk and survival, including overall and stratified estimates by sex and MetS definition. In addition to analyses using a binary definition of MetS, we also extracted continuous MetS severity scores from SRMAs, where available. Results were then stratified by severity score to examine the relationship between increasing metabolic dysfunction markers and cancer risk or survival. Estimates reported as odds ratios (ORs) were converted to relative risks (RRs) to account for differences in study design [24]. A *p*‐value of less than 0.05 was considered statistically significant, and 95% prediction intervals were calculated for the summary random effects estimates to further account for between‐study effects [25].

The *χ* ^*2*^ based Cochran Q test (p‐value < 0.1 considered significant) and the *I* ^*2*^ metric of inconsistency (> 50% considered high heterogeneity) with 95% CI were used to assess between‐study heterogeneity [26, 27].

### 2.7 Reporting bias Assessment

The Egger's regression asymmetry test was used to examine small study effects (p‐value < 0.1 considered as significant) [28]. A SRMA was considered to have a reporting bias if nonsignificant results were not adequately reported [29]. Reporting bias was determined to be at high, low, or unclear risk based on criteria specified in the Cochrane handbook [29]. Excess significance was determined by comparing the expected against the observed number of studies with statistically significant results through a chi‐square (*χ* ^2^) test (p‐value < 0.1 considered excess significance) [30, 31, 32].

### 2.8 Certainty Assessment

The quantitative umbrella review criteria, a modified version of the Ioannidis criteria, were used to stratify the strength of evidence of each association as “convincing”, “highly suggestive”, “suggestive”, “weak”, or “nonsignificant” based on the number of cases, statistically significant effect estimates, prediction intervals, small‐study effects, excess significance bias, and heterogeneity (Table 1) [33, 34, 35, 36].

**TABLE 1:** Criteria for the strength and certainty of evidence stratification.

Strength of Evidence	Criteria
Convincing (class I)	Number of cases > 1000 *p* < 10^−6^ Heterogeneity (I^2^) < 50% 95% prediction interval excluding the null No small‐study effects (*p* < 0.1) No excess significance bias (p < 0.1)
Highly suggestive (class II)	Number of cases > 1000 p < 10^−6^ Largest study with a statistically significant effect (*p* < 0.05)
Suggestive (class III)	Number of cases > 1000 p < 10^−3^
Weak (class IV)	p < 0.05
Nonsignificant	*p* > 0.05

We performed sensitivity analyses by 1) excluding original studies within each meta‐analysis that had a high risk of bias as rated by the Newcastle‐Ottawa Scale (NOS), and 2) excluding original studies within each meta‐analysis with small sample sizes (< 25th percentile) [37], and 3) analyses restricted to cohort studies (prospective and retrospective) to assess the potential influence of temporality and reverse causation.

All statistical analyses were performed using the “*meta*” (Version 6.5.0) and “*metaumbrella*” (Version 1.0.6) packages in R (The R Foundation, Vienna, Austria) [38, 39].

## 3 Results

### 3.1 Study Selection

**FIGURE 1:**
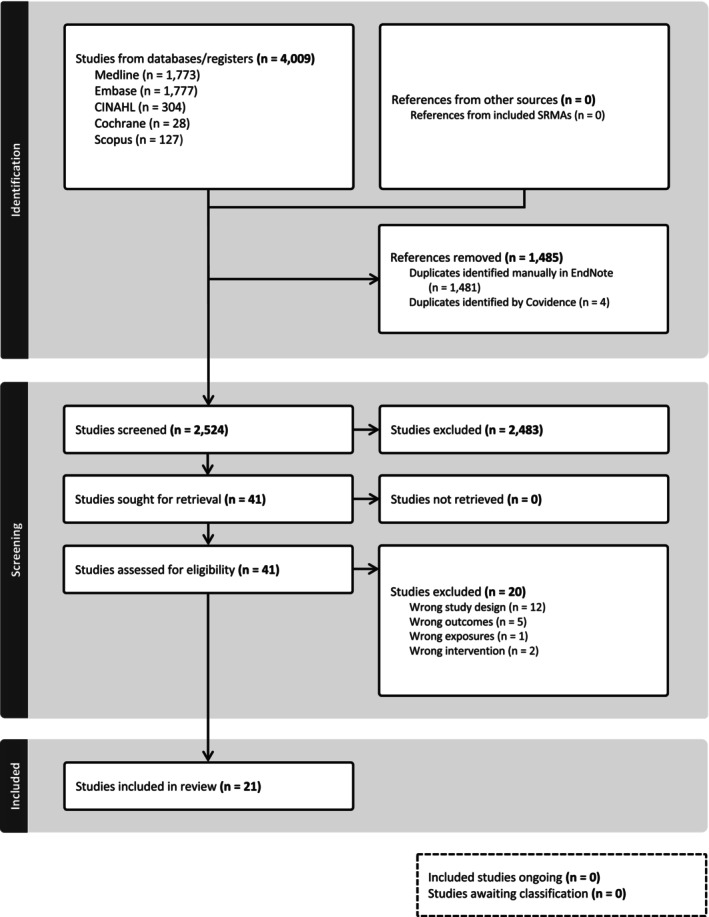
PRISMA flow diagram of the selection process of systematic reviews with meta‐analysis of metabolic syndrome and obesity‐related cancer risk and survival.

The database searches yielded 4009 Results (Figure 1). After duplicate removal, two reviewers (M.W. and P.K.) independently screened 2524 publication titles and abstracts and assessed 41 full‐text publications. After full‐text review, 21 SRMAs were included in the umbrella review (Appendix C). A bibliography of excluded full‐text articles with reasons (commonly incorrect exposure, outcomes or study design) is included in Appendix D.

### 3.2 Study Characteristics (Table 2)

**TABLE 2:** Characteristics of the systematic reviews with meta‐analysis included in the umbrella review.

Source	Cancer(s)	Included Studies	Total Sample Size^a^	Cases^a^	Reported RR(95% CI)	MetS Definition	Heterogeneity	AMSTAR 2
		**Obesity‐related Cancer Risk**						
**Esposito et al., 2012**	Breast, Colorectal, Endometrial, Liver, Ovarian, Pancreatic, Thyroid	43	Breast: 309,163 CRC: 713,800 (M 380,786; F 333,014) Endometrial: 307,556 Liver: 846,328 (M 451,569; F 411,436) Ovarian: 303,997 Pancreatic: 639,371 (M 315,723; F 323,648) Thyroid: 612,054 (M 306,543; F 305,511)	Breast: 1338 CRC: 10,021 (M 6124; F 3897) Endometrial: 2190 Liver: 4196 (M 2828; F 1368) Ovarian: 654 Pancreatic: 1294 (M 767; F 527) Thyroid: 395 (M 137; F 258)	Breast: 1.56(1.08–2.24) CRC: M 1.25(1.19–1.32); F 1.34(1.09–1.64) Endometrial: 1.40(1.32–1.49) Liver: 1.60(1.32–1.94) Ovarian: 1.26(1.00–1.59) Pancreatic: M 1.29(0.88–1.89); F 1.58(1.35–1.84) Thyroid: 1.15(0.96–1.37); 1.00(0.87–1.15)	Included traditional and non‐traditional definitions with at least 3 factors	Breast: 88% CRC: M 35%, F 60% Endometrial: 46% Liver: Overall 79%, M 80%, F 87% Ovarian: 9% Pancreatic: M 65%, F 0.0% Thyroid: M 8%, F 0%	*Critically low*
**Esposito et al., 2013**	Breast	9	375,876	7308	1.52 (1.20–1.93)	Strict and modified NCEP ATP III, ≥ 3 metabolic abnormalities, z‐scores	Overall: 85% NCEP ATP III: 63%	*Critically low*
**Esposito et al., 2013**	Colorectal	17	730,307 (M 379,162; F 351,145)	10,575 (M 6350; F 4225)	M 1.33(1.18–1.50) F 1.41(1.18–1.70)	Modified NCEP ATP III, ≥ 3 metabolic abnormalities, z‐score, quartiles, use of metabolic agents	M: 45% F: 58% NCEP ATP III: 33%	*Critically low*
**Jinjuvadia et al., 2013**	Colorectal	18	677,851	10,889	1.34 (1.24–1.44)	NCEP ATP III, IDF, AHA, WHO	51%	*Critically low*
**Bhandari et al., 2014**	Breast	8	79,101	5834	1.81 (1.28–2.56)	Strict and modified NCEP ATP III, IDF, modified IDF, modified WHO, NHLBI, J‐MeS, ≥ 3 metabolic abnormalities, tertiles, use of metabolic agents	70%	*Critically low*
**Esposito et al., 2014**	Endometrial	6	310,219	3132	1.89 (1.34–2.67)	Strict and modified NCEP ATPIII, IDF, ≥ 2 or 3 metabolic abnormalities, z‐score	92%	*Critically low*
**Jinjuvadia et al., 2014**	Liver	4	829,651	4074	1.81 (1.37–2.41)	NCEP ATP III, IDF, AHA	79%	*Critically low*
**Chen et al., 2018**	Liver	6	747,981 (M 97,912; F 88,046)	1061 (M 614; F 181)	NCEP 1.43 (1.19–1.72) IDF 1.59(1.13–2.23)	NCEP ATP III, IDF	Overall: 29% M: 65%, F: 57% IDF: 0%	*Critically low*
**Li et al., 2018**	Liver	10	887,572 (M 19,477; F 33,562)	4346 (M 113; F 56)	1.60 (1.12–2.28)	NCEP ATP III, WHO, IDF, AHA, JASSO, IASO, J‐Mes, Unknown	Overall: 90% M: 0%, F: 78%	*Critically low*
**Yin et al., 2018**	Thyroid	2	578,782	429	1.04 (0.94–1.16)	HOMA‐IR, IR, dyslipidemia, hypertension, z‐score	0%	*Critically low*
**Guo et al., 2019**	Breast	17	506,868	10,045	1.25 (1.12–1.39)	NCEP ATP III, IDF	79%	*Critically low*
**Ren et al., 2019**	Liver	19	1,566,996	2907	1.76 (1.33–2.33)	NCEP ATP III, AHA, WHO, ICD, HOMA, ADA, Asian and Chinese Criteria	88%	*Critically low*
**Wang et al., 2020**	Endometrial	6	270,052	35,193	1.50 (1.22–1.82)	Strict and modified NCEP ATP III, IDF, modified IDF	NCEP ATP III: 78% IDF: 65%	*Critically low*
**Zhao et al., 2020**	Breast	25	120,340	2130	2.01 (1.55–2.60)	Strict and modified NCEP ATP III	56%	*Critically low*
**Han et al., 2021**	Colorectal	18	23,643,370	161,280	1.25 (1.18–1.32)	Strict and modified NCEP ATP III, WHO, IDF, AHA, ≥ 3 metabolic abnormalities, use of metabolic agents	Overall: 44% NCEP: 0% IDF: 26% AHA: 57%	*Critically low*
**Shen et al., 2021**	Colorectal	21	53,010,256 (M 15,605,776; F 14,056,875)	350,817 (M 95,474; F 84,188)	1.36(1.26–1.47)	NCEP ATP III, ≥ 3 MetS components	Overall: 86%, M: 75%, F: 75% NCEP ATP III: M 72%, F 67%	*Critically low*
**Zhang et al., 2021**	Esophageal adenocarcinoma	5	793,049	3978	1.19 (1.10–1.27)	NCEP ATP III, IDF, AHA, ≥ 3 or 5 metabolic abnormalities	17%	*Critically low*
**Du et al., 2022**	Renal	8	10,601,007	32,130	1.62 (1.41–1.87)	NCEP ATP III	Overall: 85%, M: 80%, F: 77%	*Critically low*
		**Obesity‐related Cancer Survival**						
**Esposito et al., 2012**	Colorectal (CSS)	4	107,128	887	1.61 (1.28–2.01)	Traditional and non‐traditional definitions with at least 3 factors	0%	*Critically low*
**Esposito et al., 2013**	Colorectal (CSS)	5	671,704	2607	M 1.36 (1.25–1.48) F 1.16(1.03–1.3)	Modified NCEP ATP III, ≥ 3 metabolic abnormalities, z‐score, quartiles, use of metabolic agents	M: 1% F: 21%	*Critically low*
**Han et al., 2021**	Colorectal (CSS)	12	83,303	24,579	1.72 (1.03–2.42)	Strict and modified NCEP ATP III, WHO, IDF, AHA, ≥ 3 metabolic abnormalities, use of metabolic agents	OS: 85% CSS: 85%	*Critically low*
**Lu B et al., 2022**	Colorectal (CSS and OS)	2 CSS, 12 OS	CSS: 3364 OS: 116,226	CSS: 996 OS: 22,792	CSS: 2.12 (1.08–4.17) OS: 1.34(1.11–1.63)	Strict and modified NCEP ATP III, IDF, CDS, AHA, NHLBI, Harmonized, other	OS: 85% CSS: 93%	*Critically low*
**Lu L et al., 2022**	Colorectal (CSS and OS)	3 CSS, 7 OS	CSS: 4528 OS: 41,476	CSS: 889 OS: 21,752	CSS: 1.80 (1.04–3.12) OS: 1.04(0.94–1.15)	Strict and modified NCEP ATP III, CDS, AHA, ≥ 3 metabolic abnormalities	OS: 44% CSS: 93%	*Critically low*
**Li et al., 2018**	Liver (OS)	6	494	96	0.92(0.83–0.99)	NCEP ATP III, WHO, IDF, AHA, JASSO, IASO, J‐Mes, other	28%	*Critically low*
**Tao et al., 2023**	Colorectal (OS)	4	4096	2787	1.17 (0.91–1.49)	Harmonized, AHA, CDS, other	65%	*Critically low*

^a^ Male and female sample sizes may not add up to the total due to missing data in the systematic reviews or original studies.

Abbreviations: Metabolic Syndrome, MetS; Relative Risk, RR; Confidence Interval, CI; A MeaSurement Tool to Assess systematic Reviews, AMSTAR; Male, M; Female, F; National Cholesterol Education Program Adult Treatment Panel III, NCEP ATP III; International Diabetes Federation, IDF; World Health Organization, WHO; American Heart Association, AHA; American Diabetes Association, ADA; National Heart, Lung, and Blood Institute, NHLBI; Japan Society for the Study of Obesity, JASSO; Japanese definition of metabolic syndrome, J‐MeS; Chinese Diabetes Society, CDS; International Association for the Study of Obesity, IASO; colorectal, CRC; Cancer‐specific survival, CSS; overall survival, OS.

Together, the 21 SRMAs comprised a total of 98 original studies on MetS and ORC risk or survival [40, 41, 42, 43, 44, 45, 46, 47, 48, 49, 50, 51, 52, 53, 54, 55, 56, 57, 58, 59, 60]. Of the included SRMAs, 18 evaluated MetS with ORC risk, including a range of two to 43 original research studies [including cancers of postmenopausal breast (5), colorectum (5), liver (5), endometrium (3), thyroid (2), pancreas (1), ovary (1), kidney (1), and adenocarcinoma of the esophagus (1)]. Seven SRMAs evaluated MetS with ORC survival, with included survival studies ranging from two to 12 [including cancers of colorectum (6) and liver (1)]. Of these reviews, five evaluated ORC‐specific survival, and five evaluated overall survival.

The individual SRMAs included studies that utilized varying definitions of MetS (Table 2), including traditional definitions from the National Cholesterol Education Program Adult Treatment Panel III (NCEP ATP III) [61], International Diabetes Foundation (IDF) [62], and American Heart Association (AHA) [63], as well as non‐traditional definitions including self‐report of three or more metabolic abnormalities and population z‐scores or quartiles for selected biomarkers. Thirty unique associations were re‐estimated and assessed for the certainty of evidence, including 25 associations with ORC risk and 5 with survival.

### 3.3 Risk of bias (Tables S1 and S2)

Using the AMSTAR2 tool, all SRMAs had “*critically low*” quality of evidence due to poor systematic review methodology and questionable reporting for transparency and reproducibility [no a priori protocol (*n* = 19), no justification for excluded studies (*n* = 20), inadequate database searches (*n* = 14), no assessment of publication bias (*n* = 4), no bias implication discussion (*n* = 2), and incomplete risk of bias assessment (n = 1)]. Only 2 (9.5%) SRMAs reported written and published protocols, and only one SRMA provided a justification for excluded studies [40, 52, 53]. As all articles received a ‘*critically low”* rating, a subgroup analysis by AMSTAR 2 rating was not performed. However, when the criteria of protocol registration and justification for excluded studies were excluded from the AMSTAR2 assessment, most SRMAs were rated as presenting moderate to high quality evidence, with only six studies remaining in the “critically low” category. Four studies were categorized as high quality, seven as moderate quality, and six as low quality (Table S2).

### 3.4 Strength and Certainty of Evidence (Table 3, Figures 2 and 3)

**TABLE 3:** Strength and certainty of evidence of included systematic reviews with meta‐analysis evaluating metabolic syndrome with obesity‐related cancer risk or survival.

Outcome	Author, year	N studies	HR (95% CI)	*p*	Prediction interval^b^	N cases	*I* ^*2*^	Egger's test (p) ^*b*^	Excess significance (p)^b^	Largest study significant	Strength of evidence class^a^
**Risk**											
Breast	Guo et al. 2019	17	1.27 (1.12–1.43)	1.4E‐04	0.87–1.85	10,058	75%	7.2E‐03	8.0E‐06	N	III
Colorectal	Shen et al. 2021	21	1.41 (1.31–1.52)	3.0E‐19	1.04–1.91	186,123	77%	1.7E‐03	5.3E‐02	Y	II
Esophageal	Zhang et al. 2021	5	1.21 (1.02–1.43)	2.8E‐02	0.76–1.92	3978	31%	5.4E‐01	2.9E‐01	Y	IV
Endometrial	Wang et al. 2020	6	1.49 (1.23–1.80)	5.4E‐05	0.81–2.74	17,768	79%	2.6E‐02	8.1E‐02	Y	III
Liver	Ren et al. 2019	19	1.74 (1.18–2.57)	5.6E‐03	0.43–7.08	2684	89%	8.3E‐01	1.5E‐01	Y	IV
Ovarian	Esposito et al. 2012	2	1.27 (0.98–1.63)	6.6E‐02	N/A	654	88%	N/A	7.7E‐01	N	ns
Pancreatic	Esposito et al. 2012	4	1.33 (1.18–1.49)	3.3E‐06	1.02–1.72	779	54%	9.9E‐01	3.5E‐01	Y	IV
Renal	Du et al. 2022	8	1.67 (1.40–2.00)	1.9E‐08	0.98–2.85	17,025	86%	2.4E‐01	2.2E‐01	Y	II
Thyroid	Yin et al. 2018	2	1.07 (0.94–1.22)	3.3E‐01	N/A	174	0%	N/A	6.4E‐01	N	ns
**Risk: Male**											
Colorectal	Shen et al. 2021	15	1.38 (1.26–1.51)	9.3E‐12	1.03–1.84	95,474	83%	6.7E‐02	9.5E‐02	Y	II
Liver	Chen et al. 2018	5	1.41 (1.03–1.92)	3.4E‐02	0.51–3.89	614	68%	6.5E‐03	1.3E‐01	N	IV
Pancreatic	Esposito et al. 2012	4	1.26 (0.87–1.82)	2.2E‐01	0.29–5.49	767	72%	8.3E‐01	4.7E‐01	N	ns
Thyroid	Esposito et al. 2012	2	1.17 (0.88–1.56)	2.7E‐01	N/A	137	8%	N/A	N/A	N	ns
**Risk: Female**											
Colorectal	Shen et al. 2021	15	1.35 (1.19–1.53)	2.2E‐06	0.89–2.05	84,230	74%	5.5E‐03	2.5E‐05	Y	II
Liver	Chen et al. 2018	4	1.29 (0.72–2.31)	3.9E‐01	0.12–14.17	181	62%	9.4E‐01	4.8E‐01	N	ns
Pancreatic	Esposito et al. 2012	4	1.58 (1.36–1.83)	2.6E‐09	1.14–2.19	527	0%	8.7E‐01	8.3E‐01	Y	IV
Thyroid	Esposito et al. 2012	2	1.00 (0.87–1.15)	9.9E‐01	N/A	258	0%	N/A	N/A	N	ns
**Risk: NCEP**											
Breast	Esposito et al. 2013		1.81 (0.91–3.62)	9.2E‐02	0.07–46.82	593	80%	3.5E‐01	2.2E‐02	Y	ns
Colorectal	Esposito et al. 2013	6	1.43 (1.16–1.76)	9.0E‐04	0.74–2.76	2164	76%	6.4E‐01	5.2E‐01	N	III
Liver	Chen et al. 2018	6	1.35 (1.12–1.64)	2.0E‐03	0.83–2.19	1697	44%	2.7E‐01	1.4E‐01	Y	III
Endometrial	Wang et al. 2020	6	1.49 (1.23–1.80)	5.4E‐05	0.81–2.74	17,768	79%	2.6E‐02	2.7E‐05	Y	III
**Risk: IDF**											
Colorectal	Han et al. 2021	5	1.50 (1.18–1.90)	9.7E‐04	0.68–3.31	152,603	71%	9.3E‐02	4.7E‐05	Y	III
Liver	Chen et al. 2018	2	1.57 (1.02–2.40)	3.9E‐02	N/A	915	0%	N/A	N/A	N	IV
Endometrial	Wang et al. 2020	5	1.36 (1.13–1.64)	1.4E‐03	0.73–2.54	17,601	66%	2.7E‐01	3.4E‐01	N	IV
**Risk: AHA**											
Colorectal	Han et al. 2021	3	1.34 (0.94–1.90)	1.0E‐01	0.02–86.60	919	79%	6.5E‐01	7.2E‐01	N	ns
**Overall survival**											
Colorectal	Lu B et al. 2022	12	1.29 (1.07–1.57)	9.1E‐03	0.69–2.42	22,792	79%	2.3E‐02	2.0E‐03	Y	IV
Liver	Li et al. 2018	3	0.71 (0.39–1.29)	2.6E‐01	0.00–600.01	N/A	72%	3.4E‐01	N/A	N	ns
**ORC‐specific survival**											
Colorectal	Esposito et al. 2013	5	1.29 (1.12–1.48)	3.7E‐04	0.90–1.83	1000	24%	2.5E‐03	1.8E‐01	Y	IV
**ORC‐specific survival: Male**											
Colorectal	Esposito et al. 2013	5	1.33 (1.17–1.51)	1.4E‐05	0.97–1.83	1650	26%	7.5E‐03	4.7E‐05	Y	III
**ORC‐specific survival: Female**											
Colorectal	Esposito et al. 2013	4	1.20 (1.05–1.36)	7.4E‐03	0.79–1.81	957	0%	1.1E‐01	8.4E‐02	Y	IV

^a^ Strength and certainty of evidence: determined using the modified Ioannidis criteria outlined in Table 2.

^b^ Prediction intervals, Egger's test, and Excess significance test results not available for meta‐analyses with < 3 included studies.

Abbreviations: HR, hazard ratio; CI, confidence interval; N, no; Y, yes; ns, nonsignificant; NCEP, The National Cholesterol Education Program ATP III criteria; IDF, The International Diabetes Federation criteria; AHA, American Heart Association; ORC, obesity‐related cancer.

**FIGURE 2:**
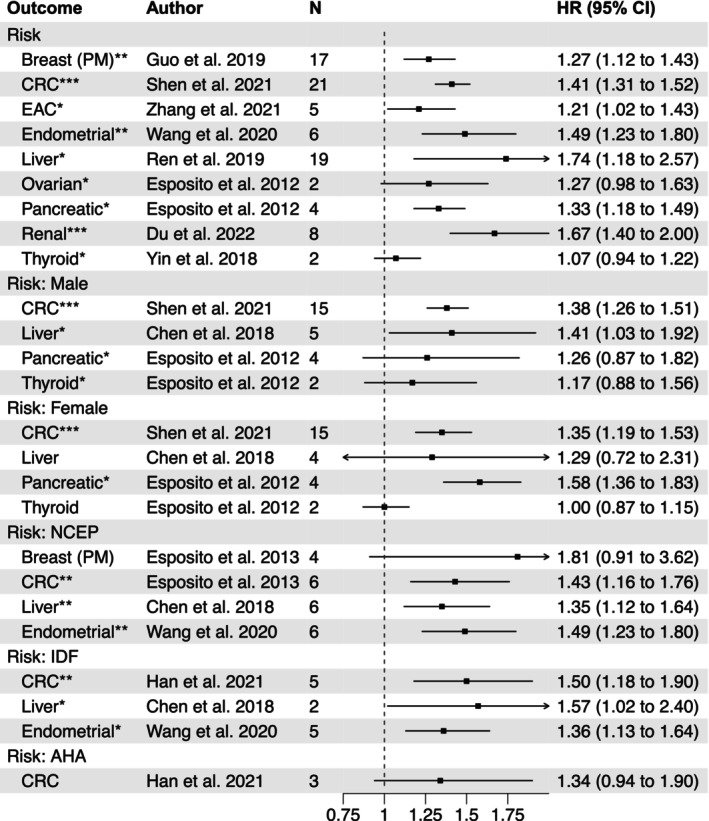
**Forest plots of the association between metabolic syndrome and obesity‐related cancer risk.** ***Highly suggestive evidence (class II), **Suggestive evidence (class III), *Weak evidence (class IV), no asterisk (nonsignificant). Determined using the modified Ioannidis criteria outlined in Table 2. Abbreviations: N, number (of studies); HR, hazard ratio; CI, confidence interval; PM, postmenopausal; CRC, colorectal cancer; EAC, esophageal adenocarcinoma cancer; NCEP, The National Cholesterol Education Program ATP III; IDF, The International Diabetes Federation; AHA, American Heart Association.

**FIGURE 3:**
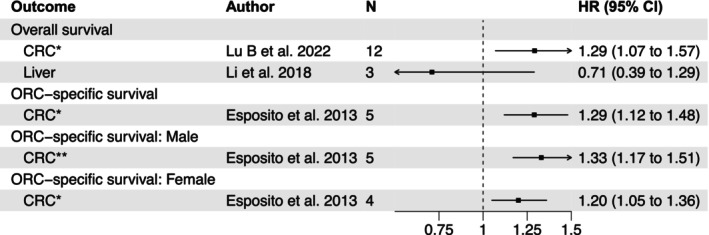
**Forest plots of the association between metabolic syndrome and obesity‐related cancer survival.** ***Highly suggestive evidence (class II), **Suggestive evidence (class III), *Weak evidence (class IV), no asterisk (nonsignificant). Determined using the modified Ioannidis criteria outlined in Table 2. Abbreviations: N, number (of studies); HR, hazard ratio; CI, confidence interval; CRC, colorectal cancer; ORC, obesity‐related cancer.

#### 3.4.1 Metabolic Syndrome and Obesity‐related Cancer Risk

Overall, 25 unique associations (unique exposure such as MetS by a specific definition, unique study population such as by sex, or unique outcome such as cancer type) evaluated MetS and ORC risk. Of these 25 associations, 13(52%) had data on more than 1000 cases and 18(72%) showed high heterogeneity (*I* ^2^ > 50%).

Nine unique associations evaluated MetS and the overall risk of ORC, including those of postmenopausal breast, colorectal, esophageal adenocarcinoma, endometrial, liver, pancreatic, and renal cancers. Of these, eight indicated modest to moderately strong associations with ORC, with hazard ratios ranging from 1.21 to 1.74. Two SRMAs were classified as ‘highly suggestive’ (colorectal and renal), two as ‘suggestive’ (postmenopausal breast and endometrial), and the rest as ‘weak’ evidence, as determined by the modified Ioannidis criteria. Two associations, including MetS with thyroid and ovarian cancers, suggested a positive association (HR 1.07 and 1.27, respectively), but neither reached statistical significance (class “ns” evidence as determined by the modified Ioannidis criteria), though each only included two original studies. Overall, 17 (68%) of the 25 associations were statistically significant and suggested increased risk.

Four unique associations evaluating MetS and ORC risk were stratified by sex. In males, the associations reported between MetS and colorectal and liver cancer risk showed a moderately increased risk [HR (CI): 1.38(1.26–1.51) and 1.41(1.03–1.92), respectively]. The association with colorectal cancer risk was classified as ‘highly suggestive’, while the association with liver cancer was classified as ‘weak’ evidence due to the small number of liver cancer cases (*n* = 614) and *p* > 0.001. The associations with pancreatic and thyroid cancers also suggested a positive association. In females, the associations between MetS and colorectal and pancreatic cancer risk reported as moderately strong evidence (HR (CI): 1.35(1.19–1.53) and 1.58(1.36–1.83), respectively). The association between MetS and the risk of colorectal cancer was classified as ‘highly suggestive’, while the risk of pancreatic cancer was classified as ‘weak’ evidence due to the small number of pancreatic cancer cases (*n* = 527). The associations with liver and thyroid cancers also suggested a positive association.

Eight unique associations evaluating MetS and the risk of an ORC were stratified by MetS definition. Associations between MetS and colorectal, endometrial, and liver cancers reported moderate evidence for these associations with HR ranging from 1.35 to 1.81 using the NCEP MetS definition. Colorectal and endometrial associations were classified as ‘suggestive’ evidence, while the association between MetS and liver cancer risk was statistically significant but classified as ‘weak’ evidence due to *p* > 0.001. When using the IDF MetS definition, the association with colorectal cancer was moderately strong [HR (CI): 1.50(1.18–1.90)] and was classified as ‘suggestive’, while the associations with liver and endometrial cancer were classified as ‘weak’ despite moderate strength (HR 1.57 and 1.36, respectively) due to p > 0.001 for both associations and a small number of liver cancer cases (*n* = 915).

#### 3.4.2 Overall Survival

Two SRMAs reported on the association between MetS and overall survival. Both associations had high heterogeneity (*I* ^*2*^ > 50%). There was a modest association between MetS and colorectal cancer overall survival [HR (CI): 1.29(1.07–1.57)], however, this association was classified as ‘weak’ evidence due to *p* > 0.001.

#### 3.4.3 Cancer‐specific Survival

Only one SRMA evaluated ORC‐specific survival (specifically, colorectal cancer) and reported results for colorectal‐specific survival and stratified by sex [45]. The association between MetS and colorectal cancer‐specific survival was classified as ‘weak’ evidence due to the number of colorectal cancer cases not being > 1000 (*n* = 1000) as determined by the modified Ioannidis criteria. However, the reported HR showed modest evidence for this association [HR (CI): 1.29(1.12–1.48)]. The association between MetS and ORC‐specific survival ranged from 1.20–1.33 in both males and females, and had low heterogeneity. The evidence in males was classified as ‘suggestive’, while the evidence in females was classified as ‘weak’ due to the low number of cases (*n* = 957) and *p* > 0.001 [HR (CI): 1.33(1.17–1.51) and 1.20(1.05–1.36), respectively].

#### 3.4.4 MetS Severity Score and ORC Risk and Survival

**FIGURE 4:**
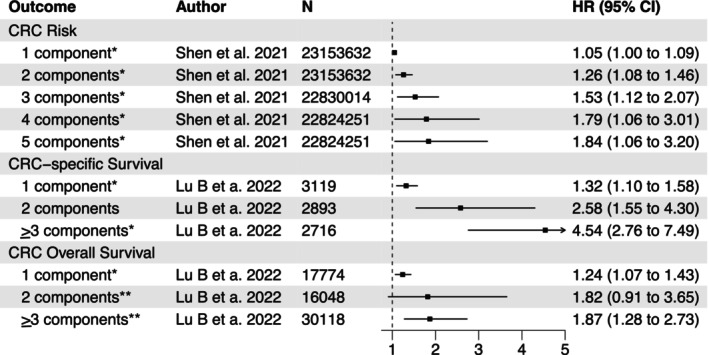
**Forest plots of the association between metabolic syndrome severity score and colorectal cancer (CRC) risk and survival.** **Suggestive evidence (class III), *Weak evidence (class IV), no asterisk (nonsignificant). Determined using the modified Ioannidis criteria outlined in Table 2. Abbreviations: N, number (of studies); HR, hazard ratio; CI, confidence interval; CRC, colorectal cancer.

Only two SRMAs reported results stratified by MetS severity score (*Shen et al*., examining MetS and colorectal cancer risk, and *Lu B et al*., examining MetS in relation to colorectal cancer‐specific and overall survival). Both studies used a continuous score based on the number of MetS components present. In these analyses, relative risks increased with a greater number of abnormal metabolic components, indicating a dose–response‐like relationship and suggesting that higher metabolic dysfunction is associated with progressively higher risk (Figure 4). While most associations were classified as ‘weak’ evidence (*p* > 0.001), the association between having two or three or more MetS components and colorectal cancer–specific survival demonstrated a 2.6‐ and 4.5‐times poorer survival and was categorized as ‘suggestive’ evidence.

In sensitivity analyses, excluding SRMAs with low quality via the NOS scale downgraded the strength of evidence of seven associations one class, one association was downgraded two classes, and one association was upgraded one class [most were downgraded from class III to IV, one was downgraded from class II to III, one was downgraded from class III to nonsignificant, and renal cancer risk was upgraded from class II to class I (convincing evidence)]. Excluding SRMAs with small sample sizes (< 25th percentile) downgraded the strength of evidence of seven associations by one class (most were downgraded from class III to IV; one was downgraded from class II to III). All associations were downgraded for increases in *p*‐value only. The remaining associations retained the same rank (data not shown). Finally, to address reverse causation, we conducted sensitivity analyses restricted to only cohort studies (including both prospective and retrospective designs). All SRMAs for survival already included only cohort studies, as did the SRMAs for liver and ovarian cancer risk, thus, these reported estimates are less susceptible to reverse causation. A sensitivity analysis was not performed for thyroid cancer risk as the SRMA only included one cohort study. For all other cancer types, analyses restricted to only cohort studies produced associations that were consistent in both direction and statistical significance with our overall results from the main analyses. The strength of evidence ratings were downgraded for breast and renal cancers due to weaker *p*‐values, and for endometrial cancer due to a reduced number of cases. However, all associations still retained the same strength of association as those not restricted to cohort studies, and the overall interpretation remained unchanged (Table S3).

### 3.5 Reporting bias (Table 3)

The Egger's and excess significance tests were significant for 8(32%) associations between MetS and ORC risk and 3(60%) associations between MetS and ORC survival, suggesting significant small study effects and selective outcome reporting and bias in the reported literature for these associations.

## 4 Discussion

In this umbrella review of observational studies, we identified 21 SRMAs reporting on 30 unique associations between MetS and ORC risk or survival. Multiple highly suggestive or suggestive associations indicated the increased risk of certain ORCs and poorer survival in individuals with MetS. However, there was significant heterogeneity among studies for most associations. This may be due to varying MetS definitions (traditional vs. non‐traditional) and study designs (case–control, cohort, cross‐sectional) of the original research studies. The stratified results by MetS definition showed large differences in ORC risk. Therefore, a uniform assessment of metabolic dysfunction may be needed to better understand its role in ORC risk and survival and allow for comparison between studies. Furthermore, despite the strength of these associations, all included SRMAs had more than one critical weakness, resulting in a “*critically low*” rating per the AMSTAR 2 criteria. Additionally, though these findings largely support a relationship between MetS and the development of most ORCs, there is still limited evidence on survival.

The connection between MetS and cancer continues to be widely studied with increases in prevalence of MetS worldwide, and there is growing evidence that MetS may play a role in the development and progression of tumors through several linked biological and lifestyle‐related factors. Biologically, MetS may lead to tumor development and progression through disruption or activation of one or multiple pathways, including gastrointestinal dysbiosis, altered adipokine and hormone release, insulin resistance, inflammation, dyslipidemia, oxidative stress, and shared behavioral risk factors [64]. Adipokines, cytokines released by adipocytes, have many functions in metabolism, cell signaling, and inflammatory pathways [65]. Specifically, decreased adiponectin has been associated with the development and progression of several cancers, as well as the development of insulin resistance, cardiovascular disease, and dyslipidemia. Because of these observations, some studies have suggested that adiponectin may be an important new marker of MetS [65]. Additionally, the excess release of leptin and resistin may lead to insulin resistance and subsequent aberrant cell proliferation and inflammation [66, 67]. TNF‐α also drives inflammation, autoimmunity, proliferation, angiogenesis, and other processes that may promote tumor development and progression [68]. Insulin resistance, a vital component of metabolic dysfunction, has been associated with the risk of several ORCs, including colorectal, gastric, pancreatic, liver, endometrial, and kidney cancer [69]. Newer evidence also suggests an association between insulin resistance and ORC survival. A recently published meta‐analysis comprised of 151 cohorts (including over 32 million individuals) observed a 1.25 times worse cancer survival in individuals with type‐2 diabetes [70].

In addition to these biological links, MetS and cancer also share common behavioral risk factors that may further influence disease development and survival, including physical activity, energy intake, and sedentary behavior [66, 71, 72, 73]. Additionally, both cancer development and cancer treatments may increase the risk of developing MetS, which may negatively impact survival [72]. MetS may further worsen survival by contributing to sarcopenia (the loss of skeletal muscle mass) and cardiovascular disease in patients with cancer [74].

A key limitation of this umbrella review is the generally low methodological quality of included SRMAs as assessed by AMSTAR 2, with the majority receiving “critically low” ratings. In most cases, these low ratings were primarily driven by the absence of pre‐registered protocols and the absence of justification for excluded studies. When these two criteria were removed from the AMSTAR2 assessment, half of the SRMAs received a high or moderate quality rating. It is important to recognize that AMSTAR 2 assigns a low quality rating if even a single critical domain is unmet, regardless of strengths in other areas (comprehensive search strategies and appropriate meta‐analytical methods). This may result in an underestimation of the potential validity and significance of findings in SRMAs that otherwise demonstrate robust methodology. Therefore, these evaluations of the methodological quality should be interpreted alongside other indicators of study quality.

In addition to this limitation, we may have missed relevant SRMAs that were not published in the English language or that were not indexed in our selected six databases. Furthermore, as this umbrella review was conducted using observational studies, there is a potential for unmeasured confounding, misclassification of MetS or ORC status, and/or reverse causality, biasing the included SRMA risk estimates. A few original studies did not report adequate sample size information, which may affect their overall weighting in the meta‐analyses. However, we expect this effect to be minimal due to the small number (*n* = 9) of original studies with missing sample sizes. Lastly, varying definitions of MetS were accepted as the exposure, which may have contributed to the high heterogeneity found in some analyses. However, this variability may reflect the broader lack of a universally accepted, standardized definition of MetS worldwide, rather than a limitation specific to this study. Consequently, differences in diagnostic criteria remain an inherent challenge in synthesizing evidence in this field. Heterogeneity in the pooled estimates may also reflect the variability in the covariates that were adjusted for in the individual studies included in the SRMAs. Despite these limitations, this review has many strengths and direct clinical relevance. To our knowledge, this is the first umbrella review conducted to systematically synthesize and assess the current evidence between MetS and ORC risk and survival. We designed and published a priori a detailed protocol that utilizes established standards (Cochrane and JBI) to reduce bias and enable transparency and reproducibility. Furthermore, we conducted this umbrella review while adhering to the PRISMA guidelines, and all protocol deviations were described and justified, further enhancing transparency. We employed a comprehensive search across different databases with robust selection methodologies. The study team for this umbrella review comprised content (MetS and ORC) and methods (epidemiologists, statisticians, librarians) experts. Additionally, including any geographic location or setting as part of our research question enhances the generalizability of these findings. The included SRMAs evaluated several ORCs and contained large numbers of both individuals with cancer and MetS. We also evaluated the quality and certainty of published evidence by using well‐established methods, including AMSTAR 2 and the modified Ioannidis criteria.

Overall, the consistent observation of increased ORC risk in individuals with MetS, supported by modest to moderate estimates and replicated across multiple SRMAs, underscores the real‐world clinical relevance of these findings. Although the strict methodological requirements of AMSTAR2 and the modified Ioannidis criteria may dampen the certainty of the evidence, the repeated observation of this association across diverse populations indicates a likely relationship between MetS and ORC and a clear need for targeted public health interventions to mitigate such an increased risk. Our results reinforce the importance of early detection and management of MetS, not only to prevent cardiovascular disease but also as a strategy for cancer prevention. Interventions that improve metabolic health, including lifestyle modification, pharmacological management of metabolic risk factors, and broader population‐level strategies to address obesity and physical inactivity, could play a crucial role in lowering the growing burden of obesity‐related cancers.

## 5 Conclusions

The results from this umbrella review indicate that metabolic syndrome (MetS) is associated with an increased risk of several obesity‐related cancers (ORCs), including breast, colorectal, esophageal, endometrial, liver, ovarian, pancreatic, and renal cancer, as well as poorer colorectal cancer survival. Although there is variability in study quality, the consistency and strength of these associations, particularly for colorectal cancer, highlight the importance of addressing MetS as a key modifiable risk factor in cancer development and progression. Public health strategies aimed at early identification, prevention, and management of MetS in the general population, as well as among individuals living with or at risk for cancer, are urgently needed.

Many of the limitations discussed in these SRMAs may be addressed by adhering to PRISMA guidelines [15, 29]. While the need for stronger, methodologically rigorous research remains, lifestyle interventions, medical management of metabolic risk factors, and policy‐level actions targeting obesity and physical inactivity should be prioritized to reduce the burden of ORCs. Randomized controlled trials aimed at improving the metabolic health of the general population and patients with cancer may be warranted to better understand the relationship between MetS and ORC risk and survival. Nonetheless, efforts to reduce metabolic dysfunction should not be delayed given the growing impact of both obesity and ORCs on global health.

## Funding

This study was supported by the following grants from the National Institute of Health: NCI K07 CA222060 (S.Hardikar), NCI R00 CA218694 (M.Playdon), NCI F30CA278348 (M.Winn), T32DK091317 NIDDK (M.Winn), NCI K00CA2644000 (P.Karra), NCI F99CA264400 (P.Karra). (The content does not necessarily represent the official views of the NIH).

## Conflicts of Interest

The authors declare no conflicts of interest.

## Supporting information


**Table S1:** Quality assessment of studies included in the umbrella review using the A MeaSurement Tool to Assess systematic Reviews (AMSTAR) 2 criteria.
**Table S2:** Quality assessment of studies included in the umbrella review using the A MeaSurement Tool to Assess systematic Reviews (AMSTAR) 2 criteria, removing two critical domains^a^. ^a^Removing two critical domains, including 1) Registered protocol prior to conducting the review, and 2) Provided justification for excluded studies.
**Table S3:** Strength and certainty of evidence including only cohort studies evaluating metabolic syndrome with obesity‐related cancer risk. Abbreviations: ORC, obesity‐related cancer; HR, hazard ratio; CI, confidence interval; PI, prediction interval; ESB, excess significance.
**Appendix A:** Deviations from the Protocol with Justifications.
**Appendix B:** Search strategy from database inception to January 03, 2023, for systematic reviews with meta‐analysis of metabolic syndrome and obesity‐related cancer risk and survival.
**Appendix C:** Bibliography of included systematic reviews with meta‐analysis.
**Appendix D**: Bibliography of excluded publications at the full‐text review stage with reasons for exclusion.

## Data Availability

Data sharing not applicable to this article as no datasets were generated or analysed during the current study.

## References

[1] Third Report of the National Cholesterol Education Program (NCEP) Expert Panel on Detection, Evaluation, and Treatment of High Blood Cholesterol in Adults (Adult Treatment Panel III) Final Report,” Circulation 106, no. 25 (2002): 3143–3421. 12485966

[2] M. Junyent , D. K. Arnett , M. Y. Tsai , et al., “Genetic Variants at the PDZ‐INTERACTING Domain of the Scavenger Receptor Class B Type I Interact With Diet to Influence the Risk of Metabolic Syndrome in Obese Men and Women,” Journal of Nutrition 139, no. 5 (2009): 842–848, 10.3945/jn.108.101196. 19321583 PMC2714388

[3] M. Garaulet and J. A. Madrid , “Chronobiology, Genetics and Metabolic Syndrome,” Current Opinion in Lipidology 20, no. 2 (2009): 127–134, 10.1097/MOL.0b013e3283292399. 19276891

[4] A. J. Lusis , A. D. Attie , and K. Reue , “Metabolic Syndrome: From Epidemiology to Systems Biology,” Nature Reviews. Genetics 9, no. 11 (2008): 819–830, 10.1038/nrg2468. PMC2829312 18852695

[5] T. Wilsgaard and B. K. Jacobsen , “Lifestyle Factors and Incident Metabolic Syndrome. The Tromso Study 1979‐2001,” Diabetes Research and Clinical Practice 78, no. 2 (2007): 217–224, 10.1016/j.diabres.2007.03.006. 17448561

[6] E. A. Molenaar , J. M. Massaro , P. F. Jacques , et al., “Association of Lifestyle Factors With Abdominal Subcutaneous and Visceral Adiposity: The Framingham Heart Study,” Diabetes Care 32, no. 3 (2009): 505–510, 10.2337/dc08-1382. 19074991 PMC2646037

[7] L. Djousse , J. A. Driver , J. M. Gaziano , J. E. Buring , and I. M. Lee , “Association Between Modifiable Lifestyle Factors and Residual Lifetime Risk of Diabetes,” Nutrition, Metabolism, and Cardiovascular Diseases 23, no. 1 (2013): 17–22, 10.1016/j.numecd.2011.08.002. PMC3274624 21982361

[8] G. Hirode and R. J. Wong , “Trends in the Prevalence of Metabolic Syndrome in the United States, 2011‐2016,” Journal of the American Medical Association 323, no. 24 (2020): 2526–2528, 10.1001/jama.2020.4501. 32573660 PMC7312413

[9] F. M. Mendonca , F. R. de Sousa , A. L. Barbosa , et al., “Metabolic Syndrome and Risk of cancer: Which link?,” Metabolism 64, no. 2 (2015): 182–189, 10.1016/j.metabol.2014.10.008. 25456095

[10] P. Karra , M. Winn , S. Pauleck , et al., “Metabolic Dysfunction and Obesity‐related cancer: Beyond Obesity and Metabolic Syndrome,” Obesity (Silver Spring) 30 (2022): 1323–1334, 10.1002/oby.23444. 35785479 PMC9302704

[11] B. Lauby‐Secretan , C. Scoccianti , D. Loomis , Y. Grosse , F. Bianchini , and K. Straif , “Body Fatness and Cancer—Viewpoint of the IARC Working Group,” New England Journal of Medicine 375 (2016): 794–798, 10.1056/NEJMsr1606602. 27557308 PMC6754861

[12] C. B. Steele , C. C. Thomas , S. J. Henley , et al., “Vital Signs: Trends in Incidence of Cancers Associated With Overweight and Obesity ‐ United States, 2005‐2014,” MMWR. Morbidity and Mortality Weekly Report 66, no. 39 (2017): 1052–1058, 10.15585/mmwr.mm6639e1. 28981482 PMC5720881

[13] R. P. Wildman , P. Muntner , K. Reynolds , et al., “The Obese without Cardiometabolic Risk Factor Clustering and the Normal Weight with Cardiometabolic Risk Factor Clustering: Prevalence and Correlates of 2 Phenotypes among the US Population (NHANES 1999–2004),” Archives of Internal Medicine 168, no. 15 (2008): 1617–1624, 10.1001/archinte.168.15.1617. 18695075

[14] A. J. Tomiyama , J. M. Hunger , J. Nguyen‐Cuu , and C. Wells , “Misclassification of Cardiometabolic Health When Using Body Mass Index Categories in NHANES 2005‐2012,” International Journal of Obesity 40, no. 5 (2016): 883–886, 10.1038/ijo.2016.17. 26841729

[15] E. Aromataris , R. Fernandez , C. Godfrey , C. Holly , H. Khalil , and P. Tungpunkom , Joanna Briggs Institute Reviewer's Manual, eds. E. Aromataris and Z. Munn (Joanna Briggs Institute, 2017:chap 10: Umbrella Reviews), https://reviewersmanual.joannabriggs.org/.

[16] M. Pollock , R. Fernandes , L. Becker , D. Pieper , and L. Hartling , Cochrane Handbook for Systematic Reviews of Interventions Version 6.0 (updated March 2020) (Cochrane, 2020), https://www.training.cochrane.org/handbook.

[17] M. J. Page , J. E. McKenzie , P. M. Bossuyt , et al., “The PRISMA 2020 Statement: An Updated Guideline for Reporting Systematic Reviews,” Systematic Reviews 10, no. 1 (2021): 89, 10.1186/s13643-021-01626-4. 33781348 PMC8008539

[18] M. L. Rethlefsen , S. Kirtley , S. Waffenschmidt , et al., “PRISMA‐S: An Extension to the PRISMA Statement for Reporting Literature Searches in Systematic Reviews,” Systematic Reviews 10, no. 1 (2021): 39, 10.1186/s13643-020-01542-z. 33499930 PMC7839230

[19] K. I. Bougioukas , E. Bouras , F. Apostolidou‐Kiouti , S. Kokkali , M. Arvanitidou , and A. B. Haidich , “Reporting Guidelines on How to Write a Complete and Transparent Abstract for Overviews of Systematic Reviews of Health Care Interventions,” Journal of Clinical Epidemiology 106 (2019): 70–79, 10.1016/j.jclinepi.2018.10.005. 30336211

[20] L. Shamseer , D. Moher , M. Clarke , et al., “Preferred Reporting Items for Systematic Review and Meta‐analysis Protocols (PRISMA‐P) 2015: Elaboration and Explanation,” BMJ 350 (2015): g7647, 10.1136/bmj.g7647. 25555855

[21] P. Karra , M. Winn , T. Casucci , et al., Metabolic Syndrome and Obesity‐Related Cancers: A Protocol for an Umbrella Review of Meta‐analyses (National Institute for Health and Care Research: PROSPERO, 2021;CRD42021230899), https://www.crd.york.ac.uk/prospero/display_record.php?ID=CRD42021230899.

[22] J. McGowan , M. Sampson , D. M. Salzwedel , E. Cogo , V. Foerster , and C. Lefebvre , “PRESS Peer Review of Electronic Search Strategies: 2015 Guideline Statement,” Journal of Clinical Epidemiology 75 (2016): 40–46, 10.1016/j.jclinepi.2016.01.021. 27005575

[23] B. J. Shea , B. C. Reeves , G. Wells , et al., “AMSTAR 2: A Critical Appraisal Tool for Systematic Reviews That Include Randomised or Non‐randomised Studies of Healthcare Interventions, or Both,” BMJ 358 (2017): j4008, 10.1136/bmj.j4008. 28935701 PMC5833365

[24] J. Zhang and K. F. Yu , “What's the Relative Risk? A Method of Correcting the Odds Ratio in Cohort Studies of Common Outcomes,” Journal of the American Medical Association 280 (1998): 1690–1691, 10.1001/jama.280.19.1690. 9832001

[25] J. P. Higgins , S. G. Thompson , and D. J. Spiegelhalter , “A Re‐evaluation of Random‐Effects Meta‐Analysis,” Journal of the Royal Statistical Society: Series A (Statistics in Society) 172, no. 1 (2009): 137–159, 10.1111/j.1467-985X.2008.00552.x. 19381330 PMC2667312

[26] J. P. Higgins , S. G. Thompson , J. J. Deeks , and D. G. Altman , “Measuring Inconsistency in Meta‐analyses,” BMJ 327, no. 7414 (2003): 557–560, 10.1136/bmj.327.7414.557. 12958120 PMC192859

[27] J. Deeks , J. Higgins , and D. Altman , “chap 10: Analysing data and undertaking meta‐analyses,” in Cochrane Handbook for Systematic Reviews of Interventions version 6.0 (Cochrane, 2023).

[28] M. Egger , G. Davey Smith , M. Schneider , and C. Minder , “Bias in Meta‐Analysis Detected by a Simple, Graphical Test,” BMJ (Clinical research ed.) 315, no. 7109 (1997): 629–634, 10.1136/bmj.315.7109.629. PMC2127453 9310563

[29] J. Higgins , J. Savović , M. Page , R. Elbers , J. Sterne , Cochrane Handbook for Systematic Reviews of Interventions Version 6.0. In: Higgins, JPT James Thomas , Chandler, J , Cumpston, M , Li, T , Page, MJ , & Welch, VA ed. Cochrane; 2023: chap 8: Assessing risk of bias in a randomized trial.

[30] K. K. Tsilidis , S. I. Papatheodorou , E. Evangelou , and J. P. Ioannidis , “Evaluation of Excess Statistical Significance in Meta‐Analyses of 98 Biomarker Associations with Cancer Risk,” Journal of the National Cancer Institute 104, no. 24 (2012): 1867–1878, 10.1093/jnci/djs437. 23090067

[31] J. P. Ioannidis , N. A. Patsopoulos , and E. Evangelou , “Uncertainty in Heterogeneity Estimates in Meta‐Analyses,” BMJ (Clinical research ed.) 335, no. 7626 (2007): 914–916, 10.1136/bmj.39343.408449.80. PMC2048840 17974687

[32] J. P. Ioannidis and T. A. Trikalinos , “An Exploratory Test for an Excess of Significant Findings,” Clinical Trials 4, no. 3 (2007): 245–253, 10.1177/1740774507079441. 17715249

[33] P. Fusar‐Poli and J. Radua , “Ten Simple Rules for Conducting Umbrella Reviews,” Evidence‐Based Mental Health 21, no. 3 (2018): 95–100, 10.1136/ebmental-2018-300014. 30006442 PMC10270421

[34] V. Bellou , L. Belbasis , I. Tzoulaki , L. T. Middleton , J. P. A. Ioannidis , and E. Evangelou , “Systematic Evaluation of the Associations Between Environmental Risk Factors and Dementia: An Umbrella Review of Systematic Reviews and Meta‐analyses,” Alzheimer's and Dementia 13, no. 4 (2017): 406–418, 10.1016/j.jalz.2016.07.152. 27599208

[35] V. Bellou , L. Belbasis , I. Tzoulaki , E. Evangelou , and J. P. Ioannidis , “Environmental Risk Factors and Parkinson's Disease: An Umbrella Review of Meta‐Analyses,” Parkinsonism & Related Disorders 23 (2016): 1–9, 10.1016/j.parkreldis.2015.12.008. 26739246

[36] L. Belbasis , V. Bellou , E. Evangelou , J. P. Ioannidis , and I. Tzoulaki , “Environmental Risk Factors and Multiple Sclerosis: An Umbrella Review of Systematic Reviews and Meta‐analyses,” Lancet Neurology 14, no. 3 (2015): 263–273, 10.1016/S1474-4422(14)70267-4. 25662901

[37] G. Wells , B. Shea , D. O'Connell , et al., The Newcastle‐Ottawa Scale (NOS) for Assessing the Quality of Nonrandomised Studies in Meta‐analyses (University of Ottawa, Department of Epidemiology and Commuunity Medicine. Accessed December 13, 2022), https://www.ohri.ca/programs/clinical_epidemiology/oxford.asp.

[38] C. A.‐O. Gosling , A. Solanes , P. Fusar‐Poli , and J. Radua , “Metaumbrella: The First Comprehensive Suite to Perform Data Analysis in Umbrella Reviews with Stratification of the Evidence,” BMJ Mental Health 26 (2023): e300534, 10.1136/bmjment-2022-300534. PMC10035783 36792173

[39] S. Balduzzi , G. Rücker , and G. Schwarzer , “How to Perform a Meta‐Analysis With R: A Practical Tutorial,” Evidence‐Based Mental Health 22, no. 4 (2019): 153–160, 10.1136/ebmental-2019-300117. 31563865 PMC10231495

[40] R. Bhandari , G. A. Kelley , T. A. Hartley , and I. R. Rockett , “Metabolic Syndrome Is Associated With Increased Breast cancer Risk: A Systematic Review With Meta‐analysis,” International Journal of Breast Cancer 2014 (2014): 189384, 10.1155/2014/189384. 25653879 PMC4295135

[41] Y. Chen , X. Li , S. Wu , W. Ye , and L. Lou , “Metabolic Syndrome and the Incidence of Hepatocellular Carcinoma: A Meta‐Analysis of Cohort Studies,” Oncotargets and Therapy 11 (2018): 6277–6285, 10.2147/ott.s154848. 30310291 PMC6166758

[42] W. Du , K. Guo , H. Jin , L. Sun , S. Ruan , and Q. Song , “Association Between Metabolic Syndrome and Risk of Renal Cell cancer: A Meta‐analysis,” Frontiers in Oncology 12 (2022): 928619, 10.3389/fonc.2022.928619. 35832547 PMC9271793

[43] K. Esposito , P. Chiodini , A. Colao , A. Lenzi , and D. Giugliano , “Metabolic Syndrome and Risk of cancer: A Systematic Review and Meta‐analysis,” Diabetes Care 35, no. 11 (2012): 2402–2411, 10.2337/dc12-0336. 23093685 PMC3476894

[44] K. Esposito , P. Chiodini , A. Capuano , et al., “Metabolic Syndrome and Postmenopausal Breast Cancer: Systematic Review and Meta‐Analysis,” Menopause (New York, N.Y.) 20, no. 12 (2013): 1301–1309, 10.1097/GME.0b013e31828ce95d. 23571527

[45] K. Esposito , P. Chiodini , A. Capuano , et al., “Colorectal cancer Association With Metabolic Syndrome and Its Components: A Systematic Review With Meta‐analysis,” Endocrine 44, no. 3 (2013): 634–647, 10.1007/s12020-013-9939-5. 23546613

[46] K. Esposito , P. Chiodini , A. Capuano , G. Bellastella , M. I. Maiorino , and D. Giugliano , “Metabolic Syndrome and Endometrial cancer: A Meta‐analysis,” Endocrine 45, no. 1 (2014): 28–36, 10.1007/s12020-013-9973-3. 23640372

[47] M. Guo , T. Liu , P. Li , et al., “Association Between Metabolic Syndrome and Breast Cancer Risk: An Updated Meta‐Analysis of Follow‐Up Studies,” Frontiers in Oncology 9 (2019): 1290, 10.3389/fonc.2019.01290. 31824862 PMC6883425

[48] F. Han , G. Wu , S. Zhang , J. Zhang , Y. Zhao , and J. Xu , “The Association of Metabolic Syndrome and Its Components With the Incidence and Survival of Colorectal cancer: A Systematic Review and Meta‐analysis,” International Journal of Biological Sciences 17, no. 2 (2021): 487–497, 10.7150/ijbs.52452. 33613107 PMC7893592

[49] R. Jinjuvadia , P. Lohia , C. Jinjuvadia , S. Montoya , and S. Liangpunsakul , “The Association Between Metabolic Syndrome and Colorectal Neoplasm: Systemic Review and Meta‐analysis,” Journal of Clinical Gastroenterology 47, no. 1 (2013): 33–44, 10.1097/MCG.0b013e3182688c15. 23090040 PMC3518571

[50] R. Jinjuvadia , S. Patel , and S. Liangpunsakul , “The Association Between Metabolic Syndrome and Hepatocellular Carcinoma: Systemic Review and Meta‐analysis,” Journal of Clinical Gastroenterology 48, no. 2 (2014): 172–177, 10.1097/MCG.0b013e3182a030c4. 24402120 PMC3887366

[51] Y. Li , J. Shi , X. Liu , Q. Deng , Y. Huang , and Z. Yang , “Metabolic Syndrome Relates to High Risk in Hepatocellular Carcinoma: A Meta‐analysis,” Discovery Medicine 26, no. 144 (2018): 185–196. 30695678

[52] B. Lu , J. Qian , and J. Li , “The Metabolic Syndrome and Its Components as Prognostic Factors in Colorectal Cancer: A Meta‐Analysis and Systematic Review,” Journal of Gastroenterology and Hepatology 38 (2022): 187–196, 10.1111/jgh.16042. 36287138 PMC10100176

[53] L. Lu , S. Koo , S. McPherson , M. A. Hull , C. J. Rees , and L. Sharp , “Systematic Review and Meta‐Analysis: Associations between Metabolic Syndrome and Colorectal Neoplasia Outcomes,” Colorectal Disease: The Official Journal of the Association of Coloproctology of Great Britain and Ireland 24 (2022): 1463–1318, 10.1111/codi.16092. 35156283

[54] H. Ren , J. Wang , Y. Gao , F. Yang , and W. Huang , “Metabolic Syndrome and Liver‐Related Events: A Systematic Review and Meta‐Analysis,” BMC Endocrine Disorders 19, no. 1 (2019): 40, 10.1186/s12902-019-0366-3. 31023282 PMC6485158

[55] X. Shen , Y. Wang , R. Zhao , et al., “Metabolic Syndrome and the Risk of Colorectal cancer: A Systematic Review and Meta‐analysis,” International Journal of Colorectal Disease 36, no. 10 (2021): 2215–2225, 10.1007/s00384-021-03974-y. 34331119

[56] W. Tao , C. Yuan , B. Kang , et al., “The Effect of Metabolic Syndrome on Colorectal cancer Prognosis After Primary Surgery,” Nutrition and Cancer 75, no. 1 (2023): 331–338, 10.1080/01635581.2022.2112243. 35976038

[57] L. Wang , Z. H. Du , J. M. Qiao , and S. Gao , “Association Between Metabolic Syndrome and Endometrial cancer Risk: A Systematic Review and Meta‐analysis of Observational Studies,” Aging 12, no. 10 (2020): 9825–9839, 10.18632/aging.103247. 32439832 PMC7288955

[58] D. T. Yin , H. He , K. Yu , et al., “The Association Between Thyroid cancer and Insulin Resistance, Metabolic Syndrome and Its Components: A Systematic Review and Meta‐analysis,” International Journal of Surgery (London, England) 57 (2018): 66–75, 10.1016/j.ijsu.2018.07.013. 30081182

[59] J. Zhang , H. Wu , and R. Wang , “Metabolic Syndrome and Esophageal Cancer Risk: A Systematic Review and Meta‐Analysis,” Diabetology and Metabolic Syndrome 13, no. 1 (2021): 8, 10.1186/s13098-021-00627-6. 33468224 PMC7816502

[60] P. Zhao , N. Xia , H. Zhang , and T. Deng , “The Metabolic Syndrome Is a Risk Factor for Breast cancer: A Systematic Review and Meta‐analysis,” Obesity Facts 13, no. 4 (2020): 384–396, 10.1159/000507554. 32698183 PMC7590763

[61] J. I. Cleeman , “Executive summary of the third report of the National Cholesterol Education Program (NCEP) expert panel on detection, evaluation, and treatment of high blood cholesterol in adults (Adult Treatment Panel III),” Journal of the American Medical Association 285, no. 19 (2001): 2486–2497, 10.1001/jama.285.19.2486. 11368702

[62] K. G. Alberti , P. Zimmet , J. Shaw , and Group IDFETFC , “The Metabolic Syndrome—A New Worldwide Definition,” Lancet 366, no. 9491 (2005): 1059–1062, 10.1016/S0140-6736(05)67402-8. 16182882

[63] S. M. Grundy , J. I. Cleeman , S. R. Daniels , et al., “Diagnosis and Management of the Metabolic Syndrome: An American Heart Association/National Heart, Lung, and Blood Institute Scientific Statement,” Circulation 112, no. 17 (2005): 2735–2752, 10.1161/circulationaha.105.169404. 16157765

[64] T. Scully , A. Ettela , D. LeRoith , and E. J. Gallagher , “Obesity, Type 2 Diabetes, and cancer Risk,” Frontiers in Oncology 10 (2020): 615375, 10.3389/fonc.2020.615375. 33604295 PMC7884814

[65] M. Chandran , S. A. Phillips , T. Ciaraldi , and R. R. Henry , “Adiponectin: More Than Just Another Fat Cell hormone?,” Diabetes Care 26, no. 8 (2003): 2442–2450, 10.2337/diacare.26.8.2442. 12882876

[66] C. M. Friedenreich , C. Ryder‐Burbidge , and J. McNeil , “Physical Activity, Obesity and Sedentary Behavior in cancer Etiology: Epidemiologic Evidence and Biologic Mechanisms,” Molecular Oncology 15, no. 3 (2021): 790–800, 10.1002/1878-0261.12772. 32741068 PMC7931121

[67] M. P. Reilly , M. Lehrke , M. L. Wolfe , A. Rohatgi , M. A. Lazar , and D. J. Rader , “Resistin is an Inflammatory Marker of Atherosclerosis in Humans,” Circulation 111, no. 7 (2005): 932–939, 10.1161/01.Cir.0000155620.10387.43. 15710760

[68] H. Lebrec , R. Ponce , B. D. Preston , J. Iles , T. L. Born , and M. Hooper , “Tumor Necrosis Factor, Tumor Necrosis Factor Inhibition, and cancer Risk,” Current Medical Research and Opinion 31, no. 3 (2015): 557–574, 10.1185/03007995.2015.1011778. 25651481

[69] S. C. Larsson , N. Spyrou , and C. S. Mantzoros , “Body Fatness Associations With cancer: Evidence From Recent Epidemiological Studies and Future Directions,” Metabolism 137 (2022): 155326, 10.1016/j.metabol.2022.155326. 36191637

[70] S. Ling , K. Brown , J. K. Miksza , et al., “Association of Type 2 Diabetes With cancer: A Meta‐analysis With bias Analysis for Unmeasured Confounding in 151 Cohorts Comprising 32 Million People,” Diabetes Care 43, no. 9 (2020): 2313–2322, 10.2337/dc20-0204. 32910779

[71] P. T. Katzmarzyk , K. E. Powell , J. M. Jakicic , R. P. Troiano , K. Piercy , and B. Tennant , “Sedentary Behavior and Health: Update From the 2018 Physical Activity Guidelines Advisory Committee,” Medicine and Science in Sports and Exercise 51, no. 6 (2019): 1227–1241, 10.1249/mss.0000000000001935. 31095080 PMC6527341

[72] E. C. de Haas , S. F. Oosting , J. D. Lefrandt , B. H. Wolffenbuttel , D. T. Sleijfer , and J. A. Gietema , “The Metabolic Syndrome in Cancer Survivors,” Lancet Oncology 11, no. 2 (2010): 193–203, 10.1016/s1470-2045(09)70287-6. 20152771

[73] E. A. Silveira , N. Kliemann , M. Noll , N. Sarrafzadegan , and C. de Oliveira , “Visceral Obesity and Incident Cancer and Cardiovascular Disease: An Integrative Review of the Epidemiological Evidence,” Obesity Reviews 22, no. 1 (2021): e13088, 10.1111/obr.13088. 32692447 PMC7757158

[74] S. J. Lee and N. C. Kim , “Association Between Sarcopenia and Metabolic Syndrome in Cancer Survivors,” Cancer Nursing 40, no. 6 (2017): 479–487, 10.1097/ncc.0000000000000454. 27922919

