# Supplementary material for: Metabolic Syndrome and Obesity‐related cancer Risk and Survival: An Umbrella Review of Systematic Reviews With Meta‐analysis of Observational Studies
Source: Obes Rev. 2026 Jan 8;27(6):e70073. doi: 10.1111/obr.70073 (PMC12795314; doi:10.1111/obr.70073)
Supplement: Supplementary file 1 — Table S1: Quality assessment of studies included in the umbrella review using the A MeaSurement Tool to Assess systematic Reviews (AMSTAR) 2 criteria. Table S2: Quality assessment of studies included in the umbrella review using the A MeaSurement Tool to Assess systematic Reviews (AMSTAR) 2 criteria, removing two critical domainsa. aRemoving two critical domains, including 1) Registered protocol prior to conducting the review, and 2) Provided justification for excluded studies. Table S3: Strength and certainty of evidence including only cohort studies evaluating metabolic syndrome with obesity‐related cancer risk. Abbreviations: ORC, obesity‐related cancer; HR, hazard ratio; CI, confidence interval; PI, prediction interval; ESB, excess significance. Appendix A: Deviations from the Protocol with Justifications. Appendix B: Search strategy from database inception to January 03, 2023, for systematic reviews with meta‐analysis of metabolic syndrome and obesity‐related cancer risk and survival. Appendix C: Bibliography of included systematic reviews with meta‐analysis. Appendix D: Bibliography of excluded publications at the full‐text review stage with reasons for exclusion. [file OBR-27-e70073-s001.pdf]

Supporting Information

Metabolic syndrome and obesity-related cancer risk and survival: An umbrella review of systematic reviews with meta-analysis of observational studies

***Maci Winn[1,2]**, *Prasoona Karra[2,3,4], Ryzen Benson[2,5,6], Svenja Pauleck[2], Nathorn Chaiyakunapruk[7], Win Khaing[7], Sajesh K. Veettil[8], Mary M. McFarland[9], Tallie Casucci[10], Yizhe Xu[11], Siwen Hu-Lieskovan[2], Michelle Litchman[12], Mary Playdon[1,2,3] and Sheetal Hardikar[1,2]

[1]University of Utah Department of Population Health Sciences, Salt Lake City, Utah, USA; [2]Huntsman Cancer Institute, Salt Lake City, Utah, USA; [3]Department of Nutrition and Integrative Physiology, University of Utah, Salt Lake City, Utah, USA; [4]Department of Epidemiology, Geisel School of Medicine at Dartmouth College, Lebanon, New Hampshire, USA; [5]University of California San Francisco Bakar Computational Health Sciences Institute, San Francisco, California, USA; [6]University of California San Francisco Department of Radiation Oncology, San Francisco, California, USA; [7]University of Utah College of Pharmacy, Salt Lake City, Utah, USA; [8]Department of Pharmacy Practice, International Medical University, Kuala Lumpur, Malaysia; [9]University of Utah Spencer S. Eccles Health Sciences Library, Salt Lake City, Utah, USA; [10]University of Utah J. Willard Marriott Library, Salt Lake City, Utah, USA; [11]University of Utah Department of Internal Medicine, Division of Epidemiology, Salt Lake City, Utah, USA, [12]University of Utah College of Nursing, Salt Lake City, Utah, USA.

Corresponding Author:
Sheetal Hardikar, MBBS, PhD, MPH
Huntsman Cancer Institute
Email: sheetal.hardikar@hci.utah.edu

Appendix A: Deviations from the Protocol with Justifications

1. Inclusion Criteria: *"MetS defined according to one of national/international health organization criteria, including: World Health Organization (WHO), National Cholesterol Education Program Adult Treatment Panel III (NCEP ATP3), International Diabetes Federation (IDF) and American Heart Association/National Heart, Lung, and Blood Institute (AHA/NHLBI)"*

At present, there is no uniform definition for Metabolic Syndrome (MetS). Additionally, observational data often has limitations in determining accurate and precise measures of metabolic biomarkers. Thus, we included systematic reviews with meta-analysis (SRMAs) that included studies with any definitions of MetS. To attempt to understand the differences between the current organizational definitions, we performed a subgroup analysis by MetS definition as available [National Cholesterol Education Program/Third Adults Treatment Panel (NCEP-ATP III), International Diabetes Federation (IDF), and American Heart Association (AHA)].

2. Strength and Certainty of Evidence: *"Finally, we will qualitatively access the overall certainty of evidence using GRADE criteria. Evidence will be categorized into high, moderate, low and very low based on 5 criteria (risk of bias, inconsistency, indirectness, imprecision and publication bias) to rate down and 3 (Large affect, dose response, all plausible residual confounding) to rate up the quality of evidence. We will develop the summary of findings table using GRADEProGDT app."*

The GRADE approach was removed from our methodology because all of SRMAs of observational studies. Instead, we used the modified Ioannidis criteria as our strength and certainty of evidence assessment.

3. Statistical analysis: *"We will perform sensitivity analyses according to different definitions of MetS as per health the organization definitions (WHO, NCEP ATP3, IDF and AHA/NHLBI) previously mentioned (22, 36, 37), for the sake of integrity and comparability with other MAs. We will also perform additional sensitivity analyses to address temporality, for example, explore the impact of excluding MA based on median follow-up time to measuring outcomes. We will investigate the sources of between-study heterogeneity by performing subgroup analysis by age (10 year intervals), sex (male and female), race/ethnicity (white and others), BMI (<25 and ≥25) and cancer site (esophagus, gastric cardia, colorectum, liver, gallbladder, pancreas, breast, uterus, ovary, kidney, meningioma, thyroid and multiple myeloma)."*

For ORC risk, stratified estimates were not reported by age group, race/ethnicity, BMI category, or follow-up time in the included SRMAs, so we were not able to evaluate the strength of evidence by these factors. For ORC survival, stratified estimates were not reported by age, race/ethnicity, BMI, MetS definition, or follow-up time in the included SRMAs, so we were not able to evaluate the strength of evidence by these factors. No SRMAs reported on the relationship between MetS and multiple myeloma, meningioma, gastric cardia, or gallbladder cancers.

Appendix B: Search strategy from database inception to January 03, 2023, for systematic reviews with meta-analysis of metabolic syndrome and obesity-related cancer risk and survival.

Initial Search Results Retrieved on February 8-11, 2021

Updated Search Results Retrieved on January 3, 2023

MEDLINE (Ovid)

1 Metabolic Syndrome/ 37397

2 (((metabolic or dysmetabolic or "insulin resistance" or reaven or cardiometabolic* or "metabolic cardiovascular") adj2 (syndrome* or "x syndrome*")) or ("deadly quartet" or "syndrome X" or ("insulin resistance" adj3 syndrome*))).ti,ab,kw. 65023

3 1 or 2 [MetS] 70559

4 Metabolic Diseases/ or Glucose Metabolism Disorders/ or ("metaboli* disease*" or "metaboli* disorder*" or "metaboli* disturbance*" or "metaboli* error*" or Thesaurismos*).ti,ab,kw. [Met Disease] 78813

5 Obesity, Abdominal/ or Abdominal Fat/ or Subcutaneous Fat, Abdominal/ or ((adipose or adiposity or fat or fatness or obesit* or obese or overweight) adj2 (abdominal* or "apple shaped" or "apple shape" or "apple type" or central or "intra abdomin*" or intraabdomin* or "intra peritoneal*" or intraperitoneal* or retroperitoneal or truncal or visceral)).ti,ab,kw. or ("waist circumference" adj1 (high* or increase* or large*)).ti,ab,kw. 46564

6 Bariatrics/ or Bariatric Medicine/ or Bariatric Surgery/ or (Bariatric* or "metabolic surger*" or "obesity surger*").ti,ab,kw. [Bariatric set] 28574

7 Dyslipidemias/ or (dyslipidemia* or dyslipidaemia* or dyslipemia* or dyslipaemia* or dyslipoproteinemia* or "dys lipidemia*" or "dys lipidaemia*" or dys-lipidemia* or dys-lipidaemia*).ti,ab,kw. 47966

8 hyperlipidemias/ or hypercholesterolemia/ or hyperlipidemia, familial combined/ or hyperlipoproteinemias/ or hyperlipoproteinemia type i/ or hyperlipoproteinemia type ii/ or hyperlipoproteinemia type iii/ or hyperlipoproteinemia type iv/ or hyperlipoproteinemia type v/ or hypertriglyceridemia/ or hypertriglyceridemic waist/ or hypolipoproteinemias/ or smith-lemli-opitz syndrome/ or lipid metabolism, inborn errors/ or (cholesteremi* or cholesterinemi* or cholesterolemi* or hypercholesterolemi* or hypercholesteremi* or hypercholesterinaemi* or hypercholesterinemi* or hypercholesterolaemi* or hyperlipidemi* or hyperlipaemi* or hyperlipoproteinemi* or "hyperotosis corticalis generalisata familiaris" or hypertriglyceridemi* or hypertriglyceridaemi* or hypolipaemi* or hypolipidaemi* or hypolipidemi* or hypolipoproteinemi*or triglyceridemi*).ti,ab,kw. or ("lipid metabolism" adj3 "inborn error*").ti,ab,kw. or ((lipemia or lipaemia) adj1 familial).ti,ab,kw. or (("smith lemli opitz" or acrodysgenital or "rutledge friedman harrod" or "rutledge lethal multiple congenital anomaly" or "rsh slo" or rsh or slo) adj1 syndrome*).ti,ab,kw. or ("dehydrocholesterol reductase" adj1 deficienc*).ti,ab,kw. 121644

9 Hyperglycemia/ or (hyperglycemi* or hyperglycaemi* or hyperglucemi* or "hyper glycemi*" or "elevated glucose blood").ti,ab,kw. 82649

10 Insulin Resistance/ or (insulin* adj2 (resistance* or response* or sensitiv*)).ti,ab,kw. 136465

11 Hypertension/ or (hypertension* or hypertensive* or ((elevated or high or raised) adj2 "blood pressure*")).ti,ab,kw. 561265

12 or/5-11 [MetS risk factors] 901767

13 breast neoplasms/ or breast carcinoma in situ/ or breast neoplasms, male/ or carcinoma, ductal, breast/ or carcinoma, lobular/ or "hereditary breast and ovarian cancer syndrome"/ or inflammatory breast neoplasms/ or triple negative breast neoplasms/ or unilateral breast neoplasms/ or colorectal neoplasms/ or adenomatous polyposis coli/ or gardner syndrome/ or colonic neoplasms/ or sigmoid neoplasms/ or colorectal neoplasms, hereditary nonpolyposis/ or rectal neoplasms/ or anus neoplasms/ or anal gland neoplasms/ or esophageal neoplasms/ or esophageal squamous cell carcinoma/ or gallbladder Neoplasms/ or kidney neoplasms/ or carcinoma, renal cell/ or wilms tumor/ or denys-drash syndrome/ or wagr syndrome/ or nephroma, mesoblastic/ or liver neoplasms/ or adenoma, liver cell/ or carcinoma, hepatocellular/ or liver neoplasms, experimental/ or Meningioma/ or multiple myeloma/ or leukemia, plasma cell/ or ovarian neoplasms/ or carcinoma, ovarian epithelial/ or granulosa cell tumor/ or luteoma/ or meigs syndrome/ or sertoli-leydig cell tumor/ or thecoma/ or pancreatic neoplasms/ or adenoma, islet cell/ or insulinoma/ or carcinoma, islet cell/ or gastrinoma/ or glucagonoma/ or somatostatinoma/ or vipoma/ or carcinoma, pancreatic ductal/ or pancreatic intraductal neoplasms/ or stomach neoplasms/ or thyroid neoplasms/ or thyroid cancer, papillary/ or thyroid nodule/ or uterine neoplasms/ or endometrial neoplasms/ or carcinoma, endometrioid/ or uterine cervical neoplasms/ or breast cyst/ or esophageal cyst/ or ovarian cysts/ or polycystic ovary syndrome/ or pancreatic cyst/ or pancreatic pseudocyst/ or parovarian cyst/ [Obesity related cancers ORC - MeSH] 1391310

14 (((cancer* or neoplasm* or adenocarcinoma* or adenoma* or blastoma* or carcinogens* or Carcinoid* or carcinoma* or carcinosarcoma* or cyst or cysts or cystic or malignant* or malignanc* or metastatic or metastases or metastasis or neoplasia or neoplastic* or oncogene* or oncogenic* or precancer* or "pre cancer*" or praecancer* or precarcinoma* or "pre carcinoma*" or praecarcinoma* or premalignan* or "pre malignan*" or polycystic* or pseudocyst* or "pseudo cyst*" or sarcoma* or tumor or tumors or tumour or tumours) adj5 ("alpha cell*" or anal or anus or "beta cell*" or breast* or cervix or cervical or "circumanal gland*" or "collecting duct*" or colon* or colorectal* or "corpus luteum" or diarrheogenic* or ductal or endometri* or esophag* or "gall bladder*" or gallbladder* or gastric* or "gastrin produc*" or "granulosa cell*" or hepatic* or hepatocellular* or hypernephroid* or "island cell*" or "islet cell*" or kidney or liver* or lobular* or mammary* or meninge* or nephroid* or oesophageal* or ovarian* or ovary* or ovaries* or pancrea* or paratubal* or parovari* or "perianal gland*" or rectal* or rectum* or renal or sigmoid* or stomach* or "theca cell*" or thyroid* or uterine* or uterus* or "vasoactive intestinal peptide*" or "vip secreting")) or androblastoma* or arrhenoblastoma* or "cardia carcinoma*" or "corpus uteri" or cystosarcoma* or "cysto sarcoma*" or dysgerminoma* or "gastric cardia" or gastrinoma* or "giant fibroadenoma" or glucagonoma* or hepatoma* or hepatoblastoma* or "hydatid* cyst* of morgagni*" or "hydatid* of morgagni* hypernephroma*" or insulinoma* or insuloma* or "kahler disease*" or "leatherbottle stomach" or "leather bottle stomach" or (leukemia* adj2 (plasmacytic* or "plasma cell*")) or "linitis plastica" or luteinoma* or luteoma* or "lynch* cancer*" or meningioma* or meningothelioma* or "morbus kahler" or "morgagni hydatid*" or (myeloma* adj2 (multiple* or multiplex* or "plasma cell*")) or "myeloma multiple*" or myelomatos* or nephroma* or nephroblastoma* or nesidioblastoma* or "paget* disease*" or "paget* nipple* disease*" or (pancrea* adj2 (cholera* or ipmn or ipmns)) or (polypos* adj2 (adenomatous or coli or colon or colus or familial or hereditary or intestinal or "myh associated")) or "sclerocystic ovar*" or somatostatinoma* or thecoma* or "thyroid nodule*" or vipoma* or "wagr comple*" or ("watery diarrhea" adj3 (hypokalem* or syndrome*))

or (("denys-drash" or drash or gardner* or grawitz* or hboc or lynch* or meig* or "Muir Torre" or phyllodes or phylloides or "polycystic ovar*" or "sertoli leydig" or "stein leventhal" or "Torre Muir" or "verner morrison" or wagr or "wagr contiguous gene" or wdha or wilm*) adj2 (tumor* or tumour* or syndrome*))).ti,ab,kw. or wdhh.ti,ab. [ORC keyword set 1] 1650004
15 (((adipose-related or "adipose tissue related" or (adipose adj2 related) or fat-related or "fat related" or "obesity-related" or "obesity related") adj6 (cancer* or neoplasm*)) or ((adipose-related or "adipose tissue related" or (adipose adj2 related) or fat-related or "fat related" or "obesity-related" or "obesity related") adj1 (adenocarcinoma* or adenoma* or blastoma* or carcinogens* or Carcinoid* or carcinoma* or carcinosarcoma* or cyst or cysts or cystic or malignant* or malignanc* or metastatic or metastases or metastasis or neoplasia or neoplastic* or oncogene* or oncogenic* or precancer* or "pre cancer*" or praecancer* or precarcinoma* or "pre carcinoma*" or praecarcinoma* or premalignan* or "pre malignan*" or polycystic* or pseudocyst* or "pseudo cyst*" or sarcoma* or tumor or tumors or tumour or tumours))).ti,ab,kw. [ORC keyword set 2] 562
16 or/13-15 [obesity-related cancers ORC] 1915348
17 meta-analysis.pt. 171404
18 meta-analysis/ or systematic review/ or meta-analysis as topic/ or "meta analysis (topic)"/ or "systematic review (topic)"/ or exp technology assessment, biomedical/ 324188
19 ("systematic review*" or (systematic* adj3 (review* or overview* or umbrella*)) or (methodologic* adj3 (review* or overview* or umbrella*)) or (review* adj2 (umbrella* or overview*))).ti,ab,kf,kw. 296556
20 ((quantitative adj3 (review* or overview* or synthes*)) or (research adj3 (integrati* or overview*))).ti,ab,kf,kw. 14702
21 ((integrative adj3 (review* or overview*)) or (collaborative adj3 (review* or overview*)) or (pool* adj3 analy*)).ti,ab,kf,kw. 36711
22 ("data synthes*" or "data extraction*" or "data abstraction*").ti,ab,kf,kw. 37513
23 (handsearch* or "hand search*").ti,ab,kf,kw. 10785
24 Statistics as Topic/ or ("mantel haenszel" or peto or "der simonian" or dersimonian or "fixed effect*" or "latin square*").ti,ab,kf,kw. 125664
25 ("met analy*" or metanaly*).ti,ab,kf,kw. 785
26 ("meta regression*" or metaregression*).ti,ab,kf,kw. 13631
27 ("meta-analy*" or metaanaly* or "systematic review*").mp,hw. 437033
28 (medline or cochrane or pubmed or medlars or embase or cinahl).ti,ab,hw. 318250
29 (cochrane or "evidence report").jw. 16368
30 or/17-29 [Final set for SR, MA -- Modified from CADTH] 705160
31 3 and 16 and 30 [MetS + ORC + SR] 288
32 (4 and 16 and 30) not 31 [Met Disease + ORC + SR] 178
33 (12 and 16 and 30) not (31 or 32) [MetS risk factors + ORC + SR] 1429
34 or/31-33 [results] 1895
35 ("25653879" or "23093685" or "23571527" or "31824862" or "23090040" or "24402120" or "32015762" or "30081182").ui. [exemplars] 8
36 34 or 35 [results include exemplars] 1895
37 remove duplicates from 36 [final results] 1773
38 limit 37 to yr="2021 -Current" 392
39 remove duplicates from 38 392

Medline (Ovid) Legend
Field codes: / = Medical Subject Heading (MeSH); ti = article title; ab = abstract; kf = keyword heading word; kw = keyword heading (author keywords); ui = unique identifier; pt = publication type; mp = multi-purpose fields; hw = subject heading word; jw = journal word
Proximity operator: adj#
Truncation: *

Embase (Embase.com)
No. Query Results
#32 #31 AND (2021:py OR 2022:py OR 2023:py) **452**
#31 #30 NOT ('conference abstract'/it OR 'conference paper'/it OR 'conference review'/it) **1,777**
#30 #27 OR #28 OR #29 **2,614**
#29 #10 AND #14 AND #26 NOT (#27 OR #28) **2,029**
#28 #2 AND #14 AND #26 NOT #27 **203**
#27 #1 AND #14 AND #26 **382**
#26 #15 OR #16 OR #17 OR #18 OR #19 OR #20 OR #21 OR #22 OR #23 OR #24 OR #25 **1,070,677**
#25 cochrane:jt OR 'evidence report':jt **24,292**
#24 'meta-analy*':de,cl OR metaanaly*:de,cl OR 'systematic review*':de,cl **565,382**
#23 'meta regression*':ti,ab,kw OR metaregression*:ti,ab,kw **16,732**
#22 'met analy*':ti,ab,kw OR metanaly*:ti,ab,kw **1,689**
#21 'statistics'/de OR 'mantel haenszel':ti,ab,kw OR peto:ti,ab,kw OR 'der simonian':ti,ab,kw OR dersimonian:ti,ab,kw OR 'fixed effect*':ti,ab,kw OR 'latin square*':ti,ab,kw **374,297**
#20 handsearch*:ti,ab,kw OR 'hand search*':ti,ab,kw **13,223**
#19 'data synthes*':ti,ab,kw OR 'data extraction*':ti,ab,kw OR 'data abstraction*':ti,ab,kw **46,643**
#18 ((integrative NEAR/3 (review* OR overview*)):ti,ab,kw) OR ((collaborative NEAR/3 (review* OR overview*)):ti,ab,kw) OR ((pool* NEAR/3 analy*):ti,ab,kw) **52,157**
#17 ((quantitative NEAR/3 (review* OR overview* OR synthes*)):ti,ab,kw) OR ((research NEAR/3 (integrati* OR overview*)):ti,ab,kw) **17,327**
#16 'systematic review*':ti,ab,kw OR ((systematic* NEAR/3 (review* OR overview* OR umbrella*)):ti,ab,kw) OR ((methodologic* NEAR/3 (review* OR overview* OR umbrella*)):ti,ab,kw) OR ((review* NEAR/2 (umbrella* OR overview*)):ti,ab,kw) **365,736**
#15 'meta analysis'/de OR 'systematic review'/de OR 'meta analysis topic'/de OR 'systematic review topic'/de OR 'systematic review (topic)'/de OR 'meta analysis (topic)'/de OR 'biomedical technology assessment'/de **579,142**
#14 #11 OR #12 OR #13 **2,429,729**
#13 ((('adipose related' OR 'adipose tissue related' OR 'adipose related' OR 'fat related' OR 'fat related' OR 'obesity-related' OR 'obesity related') NEAR/6 (cancer* OR neoplasm*)):ti,ab) OR ((('adipose related' OR 'adipose tissue related' OR 'adipose related' OR 'fat related' OR 'fat related' OR 'obesity-related' OR 'obesity related') NEAR/1 (adenocarcinoma* OR adenoma* OR blastoma* OR carcinogens* OR carcinoid* OR carcinoma* OR carcinosarcoma* OR cyst OR cysts OR cystic OR malignant* OR malignanc* OR metastatic OR metastases OR metastasis OR neoplasia OR neoplastic* OR oncogene* OR oncogenic* OR precancer* OR 'pre cancer*' OR

praecancer* OR precarcinoma* OR 'pre carcinoma*' OR praecarcinoma* OR premalignan* OR 'pre malignan*' OR polycystic* OR pseudocyst* OR 'pseudo cyst*' OR sarcoma* OR tumor OR tumors OR tumour OR tumours)):ti,ab) **898**

#12 (((cancer* OR neoplasm* OR adenocarcinoma* OR adenoma* OR blastoma* OR carcinogens* OR carcinoid* OR carcinoma* OR carcinosarcoma* OR cyst OR cysts OR cystic OR malignant* OR malignanc* OR metastatic OR metastases OR metastasis OR neoplasia OR neoplastic* OR oncogene* OR oncogenic* OR precancer* OR 'pre cancer*' OR praecancer* OR precarcinoma* OR 'pre carcinoma*' OR praecarcinoma* OR premalignan* OR 'pre malignan*' OR polycystic* OR pseudocyst* OR 'pseudo cyst*' OR sarcoma* OR tumor OR tumors OR tumour OR tumours) NEAR/5 ('alpha cell*' OR anal OR anus OR 'beta cell*' OR breast* OR cervix OR cervical OR 'circumanal gland*' OR 'collecting duct*' OR colon* OR colorectal* OR 'corpus luteum' OR diarrheogenic* OR ductal OR endometri* OR esophag* OR 'gall bladder*' OR gallbladder* OR gastric* OR 'gastrin produc*' OR 'granulosa cell*' OR hepatic* OR hepatocellular* OR hypernephroid* OR 'island cell*' OR 'islet cell*' OR kidney OR liver* OR lobular* OR mammary* OR meninge* OR nephroid* OR oesophageal* OR ovarian* OR ovary* OR ovaries* OR pancrea* OR paratubal* OR parovari* OR 'perianal gland*' OR rectal* OR rectum* OR renal OR sigmoid* OR stomach* OR 'theca cell*' OR thyroid* OR uterine* OR uterus* OR 'vasoactive intestinal peptide*' OR 'vip secreting')):ti,ab) OR androblastoma*:ti,ab OR arrhenoblastoma*:ti,ab OR 'cardia carcinoma*':ti,ab OR 'corpus uteri':ti,ab OR cystosarcoma*:ti,ab OR 'cysto sarcoma*':ti,ab OR dysgerminoma*:ti,ab OR 'gastric cardia':ti,ab OR gastrinoma*:ti,ab OR 'giant fibroadenoma':ti,ab OR glucagonoma*:ti,ab OR hepatoma*:ti,ab OR hepatoblastoma*:ti,ab OR 'hydatid* cyst* of morgagni*':ti,ab OR 'hydatid* of morgagni* hypernephroma*':ti,ab OR insulinoma*:ti,ab OR insuloma*:ti,ab OR 'kahler disease*':ti,ab OR 'leatherbottle stomach':ti,ab OR 'leather bottle stomach':ti,ab OR ((leukemia* NEAR/2 (plasmacytic* OR 'plasma cell*')):ti,ab) OR 'linitis plastica':ti,ab OR luteinoma*:ti,ab OR luteoma*:ti,ab OR 'lynch* cancer*':ti,ab OR meningioma*:ti,ab OR meningothelioma*:ti,ab OR 'morbus kahler':ti,ab OR 'morgagni hydatid*':ti,ab OR ((myeloma* NEAR/2 (multiple* OR multiplex* OR 'plasma cell*')):ti,ab) OR 'myeloma multiple*':ti,ab OR myelomatos*:ti,ab OR nephroma*:ti,ab OR nephroblastoma*:ti,ab OR nesidioblastoma*:ti,ab OR 'paget* disease*':ti,ab OR 'paget* nipple* disease*':ti,ab OR ((pancrea* NEAR/2 (cholera* OR ipmn OR ipmns)):ti,ab) OR ((polypos* NEAR/2 (adenomatous OR coli OR colon OR colus OR familial OR hereditary OR intestinal OR 'myh associated')):ti,ab) OR 'sclerocystic ovar*':ti,ab OR somatostatinoma*:ti,ab OR thecoma*:ti,ab OR 'thyroid nodule*':ti,ab OR vipoma*:ti,ab OR 'wagr comple*':ti,ab OR (('watery diarrhea' NEAR/3 (hypokalem* OR syndrome*)):ti,ab) OR ((('denys-drash' OR drash OR gardner* OR grawitz* OR hboc OR lynch* OR meig* OR 'muir torre' OR phyllodes OR phylloides OR 'polycystic ovar*' OR 'sertoli leydig' OR 'stein leventhal' OR 'torre muir' OR 'verner morrison' OR wagr OR 'wagr contiguous gene' OR wdha OR wilm*) NEAR/2 (tumor* OR tumour* OR syndrome*)):ti,ab) OR wdhh:ti,ab **2,321,619**

#11 'breast tumor'/mj OR 'breast carcinoma in situ'/mj OR 'paget nipple disease'/mj OR 'lobular carcinoma'/mj OR 'hereditary breast and ovarian cancer syndrome'/mj OR 'inflammatory breast cancer'/mj OR 'triple negative breast cancer'/mj OR 'colorectal tumor'/mj OR 'colon polyposis'/mj OR 'gardner syndrome'/mj OR 'colon tumor'/mj OR 'hereditary nonpolyposis colorectal cancer'/mj OR 'rectum tumor'/mj OR 'anus tumor'/mj OR 'esophagus tumor'/mj OR 'esophageal squamous cell carcinoma'/mj OR 'gallbladder tumor'/mj OR 'kidney tumor'/mj OR 'renal cell carcinoma'/mj OR 'nephroblastoma'/mj OR 'denys drash syndrome'/mj OR 'wagr syndrome'/mj OR 'mesoblastic nephroma'/mj OR 'liver tumor'/mj OR 'liver adenoma'/mj OR

'liver cell carcinoma'/mj OR 'mjerimental liver neoplasm' OR 'meningioma'/mj OR 'multiple myeloma'/mj OR 'plasma cell leukemia'/mj OR 'ovary tumor'/mj OR 'ovary carcinoma'/mj OR 'granulosa cell tumor'/mj OR 'luteoma'/mj OR 'meigs syndrome'/mj OR 'androblastoma'/mj OR 'thecoma'/mj OR 'pancreas tumor'/mj OR 'pancreas islet cell tumor'/mj OR 'insulinoma'/mj OR 'pancreas islet cell carcinoma'/mj OR 'gastrinoma'/mj OR 'glucagonoma'/mj OR 'somatostatinoma'/mj OR 'vipoma'/mj OR 'pancreas carcinoma'/mj OR 'intraductal papillary mucinous tumor'/mj OR 'stomach tumor'/mj OR 'thyroid tumor'/mj OR 'thyroid papillary carcinoma'/mj OR 'thyroid nodule'/mj OR 'uterus cancer'/mj OR 'endometrium tumor'/mj OR 'endometrioid carcinoma'/mj OR 'uterine cervix tumor'/mj OR 'breast cyst'/mj OR 'esophagus cyst'/mj OR 'ovary cyst'/mj OR 'ovary polycystic disease'/mj OR 'pancreas cyst'/mj OR 'pancreas pseudocyst'/mj OR 'ovary disease'/mj **674,394**

#10 #3 OR #4 OR #5 OR #6 OR #7 OR #8 OR #9 **1,235,375**

#9 'hypertension'/mj OR hypertension*:ti,ab OR hypertensive*:ti,ab OR (((elevated OR high OR raised) NEAR/2 'blood pressure*'):ti,ab) **829,885**

#8 'insulin resistance'/mj OR 'insulin response'/mj OR 'insulin sensitivity'/mj OR ((insulin* NEAR/2 (resistance* OR response* OR sensitiv*)):ti,ab) **180,606**

#7 'hyperglycemia'/mj OR hyperglycemi*:ti,ab OR hyperglycaemi*:ti,ab OR hypergluc emi*:ti,ab OR 'hyper glycemi*':ti,ab OR 'elevated glucose blood':ti,ab **109,869**

#6 'hyperlipidemia'/mj OR 'hypercholesterolemia'/mj OR 'familial hyperlipemia'/mj OR 'hyperlipoproteinemia'/mj OR 'hyperlipoproteinemia type 1'/mj OR 'hyperlipoproteinemia type 3'/mj OR 'hyperlipoproteinemia type 4'/mj OR 'hyperlipoproteinemia type 5'/mj OR 'hypertriglyceridemia'/mj OR 'hypertriglyceridemic waist'/mj OR 'lipoprotein deficiency'/mj OR 'smith lemli opitz syndrome'/mj OR 'disorders of lipid and lipoprotein metabolism'/mj OR ((cholesteremi*:ti,ab OR cholesterinemi*:ti,ab OR cholesterolemi*:ti,ab OR hypercholesterolemi*:ti,ab OR hypercholesteremi*:ti,ab OR hypercholesterinaemi*:ti,ab OR hypercholesterinemi*:ti,ab OR hypercholesterolaemi*:ti,ab OR hyperlipidemi*:ti,ab OR hyperlipaemi*:ti,ab OR hyperlipoproteinemi*:ti,ab OR 'hyperotosis corticalis generalisata familiaris':ti,ab OR hypertriglyceridemi*:ti,ab OR hypertriglyceridaemi*:ti,ab OR hypolipaemi*:ti,ab OR hypolipidaemi*:ti,ab OR hypolipidemi*:ti,ab OR hypolipoproteinemi*or:ti,ab) AND triglyceridemi*:ti,ab) OR (('lipid metabolism' NEAR/3 'inborn error*'):ti,ab) OR ((familial NEAR/1 (lipemia OR lipaemia)):ti,ab) OR ((('smith lemli opitz' OR acrodysgenital OR 'rutledge friedman harrod' OR 'rutledge lethal multiple congenital anomaly' OR 'rsh slo' OR rsh OR slo) NEAR/1 syndrome*):ti,ab) OR (('dehydrocholesterol reductase' NEAR/1 deficienc*):ti,ab) **59,343**

#5 'dyslipidemia'/mj OR dyslipidemia*:ti,ab OR dyslipidaemia*:ti,ab OR dyslipemia*:ti,ab OR dyslipaemia*:ti,ab OR dyslipoproteinemia*:ti,ab OR 'dys lipidemia*':ti,ab OR 'dys lipidaemia*':ti,ab **75,717**

#4 'bariatrics'/mj OR 'bariatric surgery'/mj OR bariatric*:ti,ab OR 'metabolic surger*':ti,ab OR 'obesity surger*':ti,ab **48,059**

#3 'abdominal obesity'/mj OR 'abdominal fat'/mj OR (((adipose OR adiposity OR fat OR fatness OR obesit* OR obese OR overweight) NEAR/2 (abdominal* OR 'apple shaped' OR 'apple shape' OR 'apple type' OR central OR 'intra abdomin*' OR intraabdomin* OR 'intra peritoneal*' OR intraperitoneal* OR retroperitoneal OR truncal OR visceral)):ti,ab) OR (('waist circumference' NEAR/1 (high* OR increase* OR large*)):ti,ab) **67,306**

#2 'metabolic disorder'/mj OR 'metaboli* disease*':ti,ab OR 'metaboli* disorder*':ti,ab OR 'metaboli* disturbance*':ti,ab OR 'metaboli* error*':ti,ab OR thesaurismos*:ti,ab **110,604**

#1 'metabolic syndrome x'/mj OR (((metabolic OR dysmetabolic OR 'insulin resistance' OR reaven OR cardiometabolic* OR 'metabolic cardiovascular') NEAR/2 (syndrome* OR 'x syndrome*')):ti,ab) OR 'deadly quartet':ti,ab OR 'syndrome x':ti,ab OR (('insulin resistance' NEAR/3 syndrome*):ti,ab) **103,211**

Embase Legend

Field codes: ti = article title; ab = abstract; kw = keyword; de = index (descriptor) term; mj = focused (descriptor) index term; cl = Embase classification; jt = source title
Proximity operator: NEAR/#
Truncation: *

CINAHL (Ebscohost)

#	Query	Limiters/Expanders	Results
S27	S23 OR S24 OR S25	Limiters - Published Date: 20210101-20231231<br>Expanders - Apply equivalent subjects<br>Search modes - Boolean/Phrase	65
S26	S23 OR S24 OR S25	Expanders - Apply equivalent subjects<br>Search modes - Boolean/Phrase	304
S25	((S5 OR S6 OR S7 OR S8 OR S9 OR S10 OR S11 OR S12 OR S13 OR S14 OR S15 OR S16 OR S17) AND (S18 OR S19 OR S20) AND (S21 OR S22)) NOT (S23 OR S24)	Expanders - Apply equivalent subjects<br>Search modes - Boolean/Phrase	235
S24	((S3 OR S4) AND (S18 OR S19 OR S20) AND (S21 OR S22)) NOT S23	Expanders - Apply equivalent subjects<br>Search modes - Boolean/Phrase	41
S23	(S1 OR S2) AND (S18 OR S19 OR S20) AND (S21 OR S22)	Expanders - Apply equivalent subjects<br>Search modes - Boolean/Phrase	28
S22	TI ("systematic review*" or (systematic* NEAR/3 (review* or overview* or umbrella*)) or (methodologic* NEAR/3 (review* or overview* or umbrella*)) or (review* NEAR/2 (umbrella* or overview*)) OR (quantitative NEAR/3 (review* or overview* or synthes*)) or (research NEAR/3	Expanders - Apply equivalent subjects<br>Search modes - Boolean/Phrase	279,624

	(integrati* or overview*)) OR (integrative NEAR/3 (review* or overview*)) or (collaborative NEAR/3 (review* or overview*)) or (pool* NEAR/3 analy*)) or "data synthes*" or "data extraction*" or "data abstraction*" or handsearch* or "hand search*" or "mantel haenszel" or peto or "der simonian" or dersimonian or "fixed effect*" or "latin square*" or "met analy*" or metanaly* or "meta regression*" or metaregression* or "meta-analy*" or metaanaly* or "systematic review*" or medline or cochrane or pubmed or medlars or embase or cinahl) OR AB ("systematic review*" or (systematic* NEAR/3 (review* or overview* or umbrella*)) or (methodologic* NEAR/3 (review* or overview* or umbrella*)) or (review* NEAR/2 (umbrella* or overview*)) OR (quantitative NEAR/3 (review* or overview* or synthes*)) or (research NEAR/3 (integrati* or overview*)) OR (integrative NEAR/3 (review* or overview*)) or (collaborative NEAR/3 (review* or overview*)) or (pool* NEAR/3 analy*)) or "data synthes*" or "data extraction*" or "data abstraction*" or handsearch* or "hand search*" or "mantel haenszel" or peto or "der simonian" or dersimonian or "fixed effect*" or "latin square*" or "met analy*" or metanaly* or "meta regression*" or metaregression* or "meta-analy*" or metaanaly* or "systematic review*" or medline or cochrane or pubmed or medlars or embase or cinahl)		
S21	(MH "Meta Analysis") or (MH "Systematic Review") OR (MH "Statistics")	Expanders - Apply equivalent subjects Search modes - Boolean/Phrase	163,066
S20	TI ((((adipose-related or "adipose tissue related" or "adipose related"or fat-related or "fat related" or "obesity-related" or "obesity related") NEAR/6 (cancer* or neoplasm*)) or ((adipose-related or "adipose tissue related" or "adipose related" or fat-related or "fat related" or "obesity-related" or "obesity related") NEAR/1 (adenocarcinoma* or adenoma* or blastoma* or carcinogens* or Carcinoid* or carcinoma* or carcinosarcoma* or cyst or cysts or cystic or malignant* or malignanc* or metastatic or metastases or metastasis or neoplasia or neoplastic* or oncogene* or oncogenic* or precancer* or "pre cancer*" or praecancer* or precarcinoma* or "pre	Expanders - Apply equivalent subjects Search modes - Boolean/Phrase	0

	carcinoma*" or praecarcinoma* or premalignan* or "pre malignan*" or polycystic* or pseudocyst* or "pseudo cyst*" or sarcoma* or tumor or tumors or tumour or tumours)))) OR AB ((((adipose-related or "adipose tissue related" or "adipose related"or fat-related or "fat related" or "obesity-related" or "obesity related") NEAR/6 (cancer* or neoplasm*)) or ((adipose-related or "adipose tissue related" or "adipose related" or fat-related or "fat related" or "obesity-related" or "obesity related") NEAR/1 (adenocarcinoma* or adenoma* or blastoma* or carcinogens* or Carcinoid* or carcinoma* or carcinosarcoma* or cyst or cysts or cystic or malignant* or malignanc* or metastatic or metastases or metastasis or neoplasia or neoplastic* or oncogene* or oncogenic* or precancer* or "pre cancer*" or praecancer* or precarcinoma* or "pre carcinoma*" or praecarcinoma* or premalignan* or "pre malignan*" or polycystic* or pseudocyst* or "pseudo cyst*" or sarcoma* or tumor or tumors or tumour or tumours))))		
S19	TI ((((cancer* or neoplasm* or adenocarcinoma* or adenoma* or blastoma* or carcinogens* or Carcinoid* or carcinoma* or carcinosarcoma* or cyst or cysts or cystic or malignant* or malignanc* or metastatic or metastases or metastasis or neoplasia or neoplastic* or oncogene* or oncogenic* or precancer* or "pre cancer*" or praecancer* or precarcinoma* or "pre carcinoma*" or praecarcinoma* or premalignan* or "pre malignan*" or polycystic* or pseudocyst* or "pseudo cyst*" or sarcoma* or tumor or tumors or tumour or tumours) NEAR/5 ("alpha cell*" or anal or anus or "beta cell*" or breast* or cervix or cervical or "circumanal gland*" or "collecting duct*" or colon* or colorectal* or "corpus luteum" or diarrheogenic* or ductal or endometri* or esophag* or "gall bladder*" or gallbladder* or gastric* or "gastrin produc*" or "granulosa cell*" or hepatic* or hepatocellular* or hypernephroid* or "island cell*" or "islet cell*" or kidney or liver* or lobular* or mammary* or meninge* or nephroid* or oesophageal* or ovarian* or ovary* or ovaries* or pancrea* or paratubal* or parovari* or "perianal gland*" or rectal* or rectum* or renal or sigmoid* or stomach* or "theca cell*" or thyroid* or uterine* or uterus* or "vasoactive	Expanders - Apply equivalent subjects Search modes - Boolean/Phrase	8,665

	intestinal peptide*" or "vip secreting")) or androblastoma* or arrhenoblastoma* or "cardia carcinoma*" or "corpus uteri" or cystosarcoma* or "cysto sarcoma*" or dysgerminoma* or "gastric cardia" or gastrinoma* or "giant fibroadenoma" or glucagonoma* or hepatoma* or hepatoblastoma* or "hydatid* cyst* of morgagni*" or "hydatid* of morgagni* hypernephroma*" or insulinoma* or insuloma* or "kahler disease*" or "leatherbottle stomach" or "leather bottle stomach" or (leukemia* NEAR/2 (plasmacytic* or "plasma cell*")) or "linitis plastica" or luteinoma* or luteoma* or "lynch* cancer*" or meningioma* or meningothelioma* or "morbus kahler" or "morgagni hydatid*" or (myeloma* NEAR/2 (multiple* or multiplex* or "plasma cell*")) or "myeloma multiple*" or myelomatos* or nephroma* or nephroblastoma* or nesidioblastoma* or "paget* disease*" or "paget* nipple* disease*" or (pancrea* NEAR/2 (cholera* or ipmn or ipmns)) or (polypos* NEAR/2 (adenomatous or coli or colon or colus or familial or hereditary or intestinal or "myh associated")) or "sclerocystic ovar*" or somatostatinoma* or thecoma* or "thyroid nodule*" or vipoma* or "wagr comple*" or ("watery diarrhea" NEAR/3 (hypokalem* or syndrome*)) or (("denys-drash" or drash or gardner* or grawitz* or hboc or lynch* or meig* or "Muir Torre" or phyllodes or phylloides or "polycystic ovar*" or "sertoli leydig" or "stein leventhal" or "Torre Muir" or "verner morrison" or wagr or "wagr contiguous gene" or wdha or wilm*) NEAR/2 (tumor* or tumour* or syndrome*)))) OR AB ((((cancer* or neoplasm* or adenocarcinoma* or adenoma* or blastoma* or carcinogens* or Carcinoid* or carcinoma* or carcinosarcoma* or cyst or cysts or cystic or malignant* or malignanc* or metastatic or metastases or metastasis or neoplasia or neoplastic* or oncogene* or oncogenic* or precancer* or "pre cancer*" or praecancer* or precarcinoma* or "pre carcinoma*" or praecarcinoma* or premalignan* or "pre malignan*" or polycystic* or pseudocyst* or "pseudo cyst*" or sarcoma* or tumor or tumors or tumour or tumours) NEAR/5 ("alpha cell*" or anal or anus or "beta cell*" or breast* or cervix or cervical or "circumanal gland*" or "collecting duct*" or colon* or colorectal* or "corpus luteum" or diarrheogenic* or ductal or		

	endometri* or esophag* or "gall bladder*" or gallbladder* or gastric* or "gastrin produc*" or "granulosa cell*" or hepatic* or hepatocellular* or hypernephroid* or "island cell*" or "islet cell*" or kidney or liver* or lobular* or mammary* or meninge* or nephroid* or oesophageal* or ovarian* or ovary* or ovaries* or pancrea* or paratubal* or parovari* or "perianal gland*" or rectal* or rectum* or renal or sigmoid* or stomach* or "theca cell*" or thyroid* or uterine* or uterus* or "vasoactive intestinal peptide*" or "vip secreting")) or androblastoma* or arrhenoblastoma* or "cardia carcinoma*" or "corpus uteri" or cystosarcoma* or "cysto sarcoma*" or dysgerminoma* or "gastric cardia" or gastrinoma* or "giant fibroadenoma" or glucagonoma* or hepatoma* or hepatoblastoma* or "hydatid* cyst* of morgagni*" or "hydatid* of morgagni* hypernephroma*" or insulinoma* or insuloma* or "kahler disease*" or "leatherbottle stomach" or "leather bottle stomach" or (leukemia* NEAR/2 (plasmacytic* or "plasma cell*")) or "linitis plastica" or luteinoma* or luteoma* or "lynch* cancer*" or meningioma* or meningothelioma* or "morbus kahler" or "morgagni hydatid*" or (myeloma* NEAR/2 (multiple* or multiplex* or "plasma cell*")) or "myeloma multiple*" or myelomatos* or nephroma* or nephroblastoma* or nesidioblastoma* or "paget* disease*" or "paget* nipple* disease*" or (pancrea* NEAR/2 (cholera* or ipmn or ipmns)) or (polypos* NEAR/2 (adenomatous or coli or colon or colus or familial or hereditary or intestinal or "myh associated")) or "sclerocystic ovar*" or somatostatinoma* or thecoma* or "thyroid nodule*" or vipoma* or "wagr comple*" or ("watery diarrhea" NEAR/3 (hypokalem* or syndrome*)) or (("denys-drash" or drash or gardner* or grawitz* or hboc or lynch* or meig* or "Muir Torre" or phyllodes or phylloides or "polycystic ovar*" or "sertoli leydig" or "stein leventhal" or "Torre Muir" or "verner morrison" or wagr or "wagr contiguous gene" or wdha or wilm*) NEAR/2 (tumor* or tumour* or syndrome*))) or) OR TI wdhh OR AB wdhh		
S18	(MH "Breast Neoplasms") OR (MH "Carcinoma, Ductal, Breast") OR (MH "Hereditary Breast and Ovarian Cancer Syndrome") OR (MH	Expanders - Apply equivalent subjects	269,083

	"Gastrointestinal Neoplasms") OR (MH "Liver Neoplasms") OR (MH "Esophageal Neoplasms") OR (MH "Intestinal Neoplasms") OR (MH "Stomach Neoplasms") OR (MH "Carcinoma, Hepatocellular") OR (MH "Esophageal Squamous Cell Carcinoma") OR (MH "Cecal Neoplasms") OR (MH "Colorectal Neoplasms") OR (MH "Duodenal Neoplasms") OR (MH "Ileal Neoplasms") OR (MH "Jejunal Neoplasms") OR (MH "Adenomatous Polyposis Coli") OR (MH "Colonic Neoplasms") OR (MH "Colorectal Neoplasms, Hereditary Nonpolyposis") OR (MH "Rectal Neoplasms") OR (MH "Gardner Syndrome") OR (MH "Sigmoid Neoplasms") OR (MH "Anus Neoplasms") OR (MH "Neoplasia, Anal Intraepithelial") OR (MH "Ovarian Neoplasms") OR (MH "Brenner Tumor") OR (MH "Carcinoma, Ovarian Epithelial") OR (MH "Thyroid Neoplasms") OR (MH "Thyroid Carcinoma, Anaplastic") OR (MH "Thyroid Nodule") OR (MH "Pancreatic Neoplasms") OR (MH "Adenoma, Islet Cell") OR (MH "Carcinoma, Islet Cell") OR (MH "Neoplasia, Pancreatic Intraepithelial") OR (MH "Insulinoma") OR (MH "Gastrinoma") OR (MH "Glucagonoma") OR (MH "Kidney Neoplasms") OR (MH "Carcinoma, Renal Cell") OR (MH "Wilms' Tumor") OR (MH "Denys-Drash Syndrome") OR (MH "WAGR Syndrome") OR (MH "Renal Cell Carcinoma, Metastatic") OR (MH "Uterine Neoplasms") OR (MH "Endometrial Neoplasms") OR (MH "Cervix Neoplasms") OR (MH "Adenocarcinoma in Situ, Cervix") OR (MH "Squamous Cell Carcinoma, Cervix") OR (MH "Ovarian Cysts") OR (MH "Esophageal Cyst") OR (MH "Polycystic Ovary Syndrome") OR (MH "Pancreatic Cyst") OR (MH "Pancreatic Pseudocyst") OR (MH "Multiple Myeloma") OR (MH "Meningioma")	Search modes - Boolean/Phrase	
S17	TI ((hypertension* or hypertensive* or ((elevated or high or raised) NEAR/2 "blood pressure*"))) OR AB ((hypertension* or hypertensive* or ((elevated or high or raised) NEAR/2 "blood pressure*")))	Expanders - Apply equivalent subjects Search modes - Boolean/Phrase	110,585
S16	(MH "Hypertension")	Expanders - Apply equivalent subjects Search modes - Boolean/Phrase	66,566

S15	TI ((insulin* NEAR/2 (resistance* or response* or sensitiv*))) OR AB ((insulin* NEAR/2 (resistance* or response* or sensitiv*)))	Expanders - Apply equivalent subjects Search modes - Boolean/Phrase	0
S14	(MH "Insulin Resistance")	Expanders - Apply equivalent subjects Search modes - Boolean/Phrase	18,252
S13	TI ((hyperglycemi* or hyperglycaemi* or hyperglucemi* or "hyper glycemi*" or "elevated glucose blood")) OR AB ((hyperglycemi* or hyperglycaemi* or hyperglucemi* or "hyper glycemi*" or "elevated glucose blood"))	Expanders - Apply equivalent subjects Search modes - Boolean/Phrase	15,413
S12	(MH "Hyperglycemia")	Expanders - Apply equivalent subjects Search modes - Boolean/Phrase	10,195
S11	TI ((cholesteremi* or cholesterinemi* or cholesterolemi* or hypercholesterolemi* or hypercholesteremi* or hypercholesterinaemi* or hypercholesterinemi* or hypercholesterolaemi* or hyperlipidemi* or hyperlipaemi* or hyperlipoproteinemi* or "hyperotosis corticalis generalisata familiaris" or hypertriglyceridemi* or hypertriglyceridaemi* or hypolipaemi* or hypolipidaemi* or hypolipidemi* or hypolipoproteinemi*or triglyceridemi*) or ("lipid metabolism" NEAR/3 "inborn error*") or (familial NEAR/1 (lipemia or lipaemia)) or (("smith lemli opitz" or acrodysgenital or "rutledge friedman harrod" or "rutledge lethal multiple congenital anomaly" or "rsh slo" or rsh or slo) NEAR/1 syndrome*) or ("dehydrocholesterol reductase" NEAR/1 deficienc*)) OR AB ((cholesteremi* or cholesterinemi* or cholesterolemi* or hypercholesterolemi* or hypercholesteremi* or hypercholesterinaemi* or hypercholesterinemi* or hypercholesterolaemi* or hyperlipidemi* or hyperlipaemi* or hyperlipoproteinemi* or "hyperotosis corticalis generalisata familiaris" or hypertriglyceridemi* or hypertriglyceridaemi* or hypolipaemi* or hypolipidaemi* or hypolipidemi* or hypolipoproteinemi*or triglyceridemi*) or ("lipid metabolism" NEAR/3 "inborn error*") or (familial	Expanders - Apply equivalent subjects Search modes - Boolean/Phrase	16,159

	NEAR/1 (lipemia or lipaemia)) or (("smith lemli opitz" or acrodysgenital or "rutledge friedman harrod" or "rutledge lethal multiple congenital anomaly" or "rsh slo" or rsh or slo) NEAR/1 syndrome*) or ("dehydrocholesterol reductase" NEAR/1 deficienc*))		
S10	(MH "Hyperlipidemia") OR (MH "Hypercholesterolemia") OR (MH "Hypercholesterolemia, Familial") OR (MH "Hyperlipoproteinemia") OR (MH "Smith-Lemli-Opitz Syndrome")	Expanders - Apply equivalent subjects Search modes - Boolean/Phrase	22,964
S9	TI ((dyslipidemia* or dyslipidaemia* or dyslipemia* or dyslipaemia* or dyslipoproteinemia* or "dys lipidemia*" or "dys lipidaemia*" or dys-lipidemia* or dys-lipidaemia*)) OR AB ((dyslipidemia* or dyslipidaemia* or dyslipemia* or dyslipaemia* or dyslipoproteinemia* or "dys lipidemia*" or "dys lipidaemia*" or dys-lipidemia* or dys-lipidaemia*))	Expanders - Apply equivalent subjects Search modes - Boolean/Phrase	10,190
S8	TI (Bariatric* or "metabolic surger*" or "obesity surger*") OR AB (Bariatric* or "metabolic surger*" or "obesity surger*")	Expanders - Apply equivalent subjects Search modes - Boolean/Phrase	8,331
S7	(MH "Bariatric Surgery") OR (MH "Bariatric Patients")	Expanders - Apply equivalent subjects Search modes - Boolean/Phrase	6,785
S6	TI (((adipose or adiposity or fat or fatness or obesit* or obese or overweight) NEAR/2 (abdominal* or "apple shaped" or "apple shape" or "apple type" or central or "intra abdomin*" or intraabdomin* or "intra peritoneal*" or intraperitoneal* or retroperitoneal or truncal or visceral)) or ("waist circumference" NEAR/1 (high* or increase* or large*))) OR AB (((adipose or adiposity or fat or fatness or obesit* or obese or overweight) NEAR/2 (abdominal* or "apple shaped" or "apple shape" or "apple type" or central or "intra abdomin*" or intraabdomin* or "intra peritoneal*" or intraperitoneal* or retroperitoneal or truncal or visceral)) or ("waist circumference" NEAR/1 (high* or increase* or large*)))	Expanders - Apply equivalent subjects Search modes - Boolean/Phrase	0
S5	(MH "Abdominal Fat")	Expanders - Apply equivalent subjects Search modes - Boolean/Phrase	2,117

S4	TI (("metaboli* disease*" or "metaboli* disorder*" or "metaboli* disturbance*" or "metaboli* error*" or Thesaurismos*)) OR AB (("metaboli* disease*" or "metaboli* disorder*" or "metaboli* disturbance*" or "metaboli* error*" or Thesaurismos*))	Expanders - Apply equivalent subjects Search modes - Boolean/Phrase	10,168
S3	(MH "Metabolic Diseases")	Expanders - Apply equivalent subjects Search modes - Boolean/Phrase	6,087
S2	TI ((((metabolic or dysmetabolic or "insulin resistance" or reaven or cardiometabolic* or "metabolic cardiovascular") NEAR/2 (syndrome* or "x syndrome*")) or ("deadly quartet" or "syndrome X" or ("insulin resistance" NEAR/3 syndrome*)))) OR AB ((((metabolic or dysmetabolic or "insulin resistance" or reaven or cardiometabolic* or "metabolic cardiovascular") NEAR/2 (syndrome* or "x syndrome*")) or ("deadly quartet" or "syndrome X" or ("insulin resistance" NEAR/3 syndrome*))))	Expanders - Apply equivalent subjects Search modes - Boolean/Phrase	366
S1	(MH "Metabolic Syndrome X")	Expanders - Apply equivalent subjects Search modes - Boolean/Phrase	13,099

CINAHL Legend
Field codes: TI = title; AB= abstract; MH = CINAHL Exact Subject Headings
Proximity operator: N#
Truncation: *

Cochrane Library (Wiley)
ID Search Hits
#1 MeSH descriptor: [Metabolic Syndrome] this term only 2138
#2 (((metabolic or dysmetabolic or "insulin resistance" or reaven or cardiometabolic* or "metabolic cardiovascular") NEAR/2 (syndrome* or "x syndrome*")) or ("deadly quartet" or "syndrome X" or ("insulin resistance" NEAR/3 syndrome*))):ti,ab,kw 8517
#3 #1 or #2 8517
#4 MeSH descriptor: [Metabolic Diseases] this term only 570
#5 MeSH descriptor: [Glucose Metabolism Disorders] this term only 173
#6 ("metabolic disease*" or "metabolic disorder*" or "metabolic disturbance*" or "metabolic error*" or Thesaurismos*):ti,ab,kw 2603
#7 #4 or #5 or #6 3174
#8 MeSH descriptor: [Obesity, Abdominal] this term only 445
#9 MeSH descriptor: [Abdominal Fat] this term only 192
#10 MeSH descriptor: [Subcutaneous Fat, Abdominal] this term only 69

#11 ((adipose or adiposity or fat or fatness or obesit* or obese or overweight) NEAR/2 (abdominal* or "apple shaped" or "apple shape" or "apple type" or central or "intra abdomin*" or intraabdomin* or "intra peritoneal*" or intraperitoneal* or retroperitoneal or truncal or visceral)):ti,ab or ("waist circumference" NEAR/1 (high* or increase* or large*)):ti,ab 5958
#12 MeSH descriptor: [Bariatrics] this term only 6
#13 MeSH descriptor: [Bariatric Medicine] this term only 0
#14 MeSH descriptor: [Bariatric Surgery] this term only 400
#15 (Bariatric* or "metabolic surger*" or "obesity surger*"):ti,ab,kw 3243
#16 MeSH descriptor: [Dyslipidemias] this term only 1390
#17 (dyslipidemia* or dyslipidaemia* or dyslipemia* or dyslipaemia* or dyslipoproteinemia* or "dys lipidemia*" or "dys lipidaemia*" or dys-lipidemia* or dys-lipidaemia*):ti,ab,kw 6746
#18 MeSH descriptor: [Hyperlipidemias] this term only 2067
#19 MeSH descriptor: [Hypercholesterolemia] this term only 3620
#20 MeSH descriptor: [Hyperlipidemia, Familial Combined] this term only 86
#21 MeSH descriptor: [Hyperlipoproteinemias] this term only 539
#22 MeSH descriptor: [Hyperlipoproteinemia Type I] this term only 20
#23 MeSH descriptor: [Hyperlipoproteinemia Type II] this term only 540
#24 MeSH descriptor: [Hyperlipoproteinemia Type III] this term only 19
#25 MeSH descriptor: [Hyperlipoproteinemia Type IV] this term only 54
#26 MeSH descriptor: [Hyperlipoproteinemia Type V] this term only 41
#27 MeSH descriptor: [Hypertriglyceridemia] this term only 705
#28 MeSH descriptor: [Hypertriglyceridemic Waist] this term only 6
#29 MeSH descriptor: [Hypolipoproteinemias] this term only 12
#30 MeSH descriptor: [Smith-Lemli-Opitz Syndrome] this term only 6
#31 MeSH descriptor: [Lipid Metabolism, Inborn Errors] this term only39
#32 (cholesteremi* or cholesterinemi* or cholesterolemi* or hypercholesterolemi* or hypercholesteremi* or hypercholesterinaemi* or hypercholesterinemi* or hypercholesterolaemi* or hyperlipidemi* or hyperlipaemi* or hyperlipoproteinemi* or "hyperotosis corticalis generalisata familiaris" or hypertriglyceridemi* or hypertriglyceridaemi* or hypolipaemi* or hypolipidaemi* or hypolipidemi* or hypolipoproteinemi* or triglyceridemi*):ti,ab,kw or ("lipid metabolism" NEAR/3"inborn error*"):ti,ab,kw or ((lipemia or lipaemia) NEAR/1 familial):ti,ab,kw or (("smith lemli opitz" or acrodysgenital or "rutledge friedman harrod" or "rutledge lethal multiple congenital anomaly" or "rsh slo" or rsh or slo) NEAR/1 syndrome*):ti,ab,kw or ("dehydrocholesterol reductase" NEAR/1 deficienc*):ti,ab,kw 17565
#33 MeSH descriptor: [Hyperglycemia] this term only 2084
#34 (hyperglycemi* or hyperglycaemi* or hyperglucemi* or "hyper glycemi*" or "elevated glucose blood"):ti or (hyperglycemi* or hyperglycaemi* or hyperglucemi* or "hyper glycemi*" or "elevated glucose blood"):ab 7957
#35 MeSH descriptor: [Insulin Resistance] this term only 5411
#36 (insulin* adj2 (resistance* or response* or sensitiv*)):ti or (insulin* adj2 (resistance* or response* or sensitiv*)):ab 0
#37 MeSH descriptor: [Hypertension] this term only 18969
#38 (hypertension* or hypertensive* or ((elevated or high or raised) adj2 "blood pressure*")):ti or (hypertension* or hypertensive* or ((elevated or high or raised) adj2 "blood pressure*")):ab 62369

#39 #8 or #9 or #10 or #11 or #12 or #13 or #14 or #15 or #16 or #17 or #18 or #19 or #20 or #21 or #22 or #23 or #24 or #25 or #26 or #27 or #28 or #29 or #30 or #31 or #32 or #33 or #34 or #35 or #36 or #37 or #38 102228
#40 MeSH descriptor: [Breast Neoplasms] this term only 14735
#41 MeSH descriptor: [Breast Carcinoma In Situ] this term only48
#42 MeSH descriptor: [Breast Neoplasms, Male] this term only 62
#43 MeSH descriptor: [Carcinoma, Ductal, Breast] this term only 378
#44 MeSH descriptor: [Carcinoma, Lobular] this term only 176
#45 MeSH descriptor: [Hereditary Breast and Ovarian Cancer Syndrome] this term only 30
#46 MeSH descriptor: [Inflammatory Breast Neoplasms] this term only 29
#47 MeSH descriptor: [Triple Negative Breast Neoplasms] this term only 380
#48 MeSH descriptor: [Unilateral Breast Neoplasms] this term only 17
#49 MeSH descriptor: [Colorectal Neoplasms] this term only 6013
#50 MeSH descriptor: [Adenomatous Polyposis Coli] this term only 129
#51 MeSH descriptor: [Gardner Syndrome] this term only 1
#52 MeSH descriptor: [Colonic Neoplasms] this term only 1880
#53 MeSH descriptor: [Sigmoid Neoplasms] this term only 75
#54 MeSH descriptor: [Colorectal Neoplasms, Hereditary Nonpolyposis] this term only 74
#55 MeSH descriptor: [Rectal Neoplasms] this term only 1938
#56 MeSH descriptor: [Anus Neoplasms] this term only 141
#57 MeSH descriptor: [Anal Gland Neoplasms] this term only 0
#58 MeSH descriptor: [Esophageal Neoplasms] this term only 1818
#59 MeSH descriptor: [Esophageal Squamous Cell Carcinoma] this term only 173
#60 MeSH descriptor: [Gallbladder Neoplasms] this term only 97
#61 MeSH descriptor: [Kidney Neoplasms] this term only 1026
#62 MeSH descriptor: [Carcinoma, Renal Cell] this term only 1067
#63 MeSH descriptor: [Wilms Tumor] this term only 84
#64 MeSH descriptor: [Denys-Drash Syndrome] this term only 0
#65 MeSH descriptor: [WAGR Syndrome] this term only 0
#66 MeSH descriptor: [Nephroma, Mesoblastic] this term only 2
#67 MeSH descriptor: [Liver Neoplasms] this term only 2678
#68 MeSH descriptor: [Adenoma, Liver Cell] this term only 3
#69 MeSH descriptor: [Carcinoma, Hepatocellular] this term only 2061
#70 MeSH descriptor: [Liver Neoplasms, Experimental] this term only 3
#71 MeSH descriptor: [Meningioma] this term only 77
#72 MeSH descriptor: [Multiple Myeloma] this term only 1819
#73 MeSH descriptor: [Leukemia, Plasma Cell] this term only 3
#74 MeSH descriptor: [Ovarian Neoplasms] this term only 2157
#75 MeSH descriptor: [Carcinoma, Ovarian Epithelial] this term only 347
#76 MeSH descriptor: [Granulosa Cell Tumor] this term only 3
#77 MeSH descriptor: [Luteoma] this term only 0
#78 MeSH descriptor: [Meigs Syndrome] this term only 0
#79 MeSH descriptor: [Sertoli-Leydig Cell Tumor] this term only 1
#80 MeSH descriptor: [Thecoma] this term only 0

#81 MeSH descriptor: [Pancreatic Neoplasms] this term only 1989
#82 MeSH descriptor: [Adenoma, Islet Cell] this term only 30
#83 MeSH descriptor: [Insulinoma] this term only 14
#84 MeSH descriptor: [Carcinoma, Islet Cell] this term only 8
#85 MeSH descriptor: [Gastrinoma] this term only 7
#86 MeSH descriptor: [Glucagonoma] this term only 2
#87 MeSH descriptor: [Somatostatinoma] this term only 3
#88 MeSH descriptor: [Vipoma] this term only 2
#89 MeSH descriptor: [Carcinoma, Pancreatic Ductal] this term only 114
#90 MeSH descriptor: [Pancreatic Intraductal Neoplasms] this term only 3
#91 MeSH descriptor: [Stomach Neoplasms] this term only 2936
#92 MeSH descriptor: [Thyroid Neoplasms] this term only 592
#93 MeSH descriptor: [Thyroid Cancer, Papillary] this term only 53
#94 MeSH descriptor: [Thyroid Nodule] this term only 156
#95 MeSH descriptor: [Uterine Neoplasms] this term only 785
#96 MeSH descriptor: [Endometrial Neoplasms] this term only 671
#97 MeSH descriptor: [Carcinoma, Endometrioid] this term only 85
#98 MeSH descriptor: [Uterine Cervical Neoplasms] this term only 2363
#99 MeSH descriptor: [Breast Cyst] this term only 2
#100 MeSH descriptor: [Esophageal Cyst] this term only 0
#101 MeSH descriptor: [Ovarian Cysts] this term only 144
#102 MeSH descriptor: [Polycystic Ovary Syndrome] this term only 1741
#103 MeSH descriptor: [Pancreatic Cyst] this term only 24
#104 MeSH descriptor: [Pancreatic Pseudocyst] this term only 29
#105 MeSH descriptor: [Parovarian Cyst] this term only 2
#106 (((cancer* or neoplasm* or adenocarcinoma* or adenoma* or blastoma* or carcinogens* or Carcinoid* or carcinoma* or carcinosarcoma* or cyst or cysts or cystic or malignant* or malignanc* or metastatic or metastases or metastasis or neoplasia or neoplastic* or oncogene* or oncogenic* or precancer* or "pre cancer*" or praecancer* or precarcinoma* or "pre carcinoma*" or praecarcinoma* or premalignan* or "pre malignan*" or polycystic* or pseudocyst* or "pseudo cyst*" or sarcoma* or tumor or tumors or tumour or tumours) NEAR/5 ("alpha cell*" or anal or anus or "beta cell*" or breast* or cervix or cervical or "circumanal gland*" or "collecting duct*" or colon* or colorectal* or "corpus luteum" or diarrheogenic* or ductal or endometri* or esophag* or "gall bladder*" or gallbladder* or gastric* or "gastrin produc*" or "granulosa cell*" or hepatic* or hepatocellular* or hypernephroid* or "island cell*" or "islet cell*" or kidney or liver* or lobular* or mammary* or meninge* or nephroid* or oesophageal* or ovarian* or ovary* or ovaries* or pancrea* or paratubal* or parovari* or "perianal gland*" or rectal* or rectum* or renal or sigmoid* or stomach* or "theca cell*" or thyroid* or uterine* or uterus* or "vasoactive intestinal peptide*" or "vip secreting")) or androblastoma* or arrhenoblastoma* or "cardia carcinoma*" or "corpus uteri" or cystosarcoma* or "cysto sarcoma*" or dysgerminoma* or "gastric cardia" or gastrinoma* or "giant fibroadenoma" or glucagonoma* or hepatoma* or hepatoblastoma* or "hydatid* cyst* of morgagni*" or "hydatid* of morgagni* hypernephroma*" or insulinoma* or insuloma* or "kahler disease*" or "leatherbottle stomach" or "leather bottle stomach" or (leukemia* NEAR/2 (plasmacytic* or "plasma cell*")) or "linitis plastica" or luteinoma* or luteoma* or "lynch* cancer*" or meningioma* or meningothelioma* or "morbus kahler" or "morgagni hydatid*" or (myeloma* NEAR/2 (multiple* or multiplex* or "plasma

cell*")) or "myeloma multiple*" or myelomatos* or nephroma* or nephroblastoma* or nesidioblastoma* or "paget* disease*" or "paget* nipple* disease*" or (pancrea* NEAR/2 (cholera* or ipmn or ipmns)) or (polypos* NEAR/2 (adenomatous or coli or colon or colus or familial or hereditary or intestinal or "myh associated")) or "sclerocystic ovar*" or somatostatinoma* or thecoma* or "thyroid nodule*" or vipoma* or "wagr comple*" or ("watery diarrhea" NEAR/3 (hypokalem* or syndrome*)) or (("denys-drash" or drash or gardner* or grawitz* or hboc or lynch* or meig* or "Muir Torre" or phyllodes or phylloides or "polycystic ovar*" or "sertoli leydig" or "stein leventhal" or "Torre Muir" or "verner morrison" or wagr or "wagr contiguous gene" or wdha or wilm*) NEAR/2 (tumor* or tumour* or syndrome*))):ti,ab,kw or wdhh:ti,ab,kw 132352
#107 (((adipose-related or "adipose tissue related" or (adipose NEAR/2 related) or fat-related or "fat related" or "obesity-related" or "obesity related") NEAR/6 (cancer* or neoplasm*)) or ((adipose-related or "adipose tissue related" or (adipose NEAR/2 related) or fat-related or "fat related" or "obesity-related" or "obesity related") NEAR/1 (adenocarcinoma* or adenoma* or blastoma* or carcinogens* or Carcinoid* or carcinoma* or carcinosarcoma* or cyst or cysts or cystic or malignant* or malignanc* or metastatic or metastases or metastasis or neoplasia or neoplastic* or oncogene* or oncogenic* or precancer* or "pre cancer*" or praecancer* or precarcinoma* or "pre carcinoma*" or praecarcinoma* or premalignan* or "pre malignan*" or polycystic* or pseudocyst* or "pseudo cyst*" or sarcoma* or tumor or tumors or tumour or tumours))):ti,ab,kw 44
#108 #40 or #41 or #42 or #43 or #44 or #45 or #46 or #47 or #48 or #49 or #50 or #51 or #52 or #53 or #54 or #55 or #56 or #57 or #58 or #59 or #60 or #61 or #62 or #63 or #64 or #65 or #66 or #67 or #68 or #69 or #70 or #71 or #72 or #73 or #74 or #75 or #76 or #77 or #78 or #79 or #80 or #81 or #82 or #83 or #84 or #85 or #86 or #87 or #88 or #89 or #90 or #91 or #92 or #93 or #94 or #95 or #96 or #97 or #98 or #99 or #100 or #101 or #102 or #103 or #104 or #105 or #106 or #107 132366
#109 #3 AND #108 429
#110 #7 AND #108 233
#111 #39 AND #108 3885
#112 #109 or #110 or #111 4335
Initial Results: 27 reviews on February 11, 2021
Updated Results: 1 review on January 3, 2023
Total Limited to Cochrane Reviews: 28

Cochrane Library Legend
Field codes: ti = Title; ab = Abstract; kw = Keyword; MeSH descriptor = Medical Subject Heading
Proximity operator: NEAR/#
Truncation: *

Scopus (scopus.com)
(TITLE-ABS (((metabolic OR dysmetabolic OR "insulin resistance" OR reaven OR cardiometabolic* OR "metabolic cardiovascular") W/2 (syndrome* OR "x syndrome*")) OR "deadly quartet" OR "syndrome X" OR ("insulin resistance" W/3 syndrome*) OR "metaboli* disease*" OR "metaboli* disorder*" OR "metaboli* disturbance*" OR "metaboli* error*" OR thesaurismos* OR ((adipose OR adiposity OR fat OR fatness OR

obesit* OR obese OR overweight) W/2 (abdominal* OR "apple shaped" OR "apple shape" OR "apple type" OR central OR "intra abdomin*" OR intraabdomin* OR "intra peritoneal*" OR intraperitoneal* OR retroperitoneal OR truncal OR visceral)) OR ("waist circumference" W/1 (high* OR increase* OR large*)) OR bariatric* OR "metabolic surger*" OR "obesity surger*" OR dyslipidemia* OR dyslipidaemia* OR dyslipemia* OR dyslipaemia* OR dyslipoproteinemia* OR "dys lipidemia*" OR "dys lipidaemia*" OR dys-lipidemia* OR dys-lipidaemia* OR cholesteremi* OR cholesterinemi* OR cholesterolemi* OR hypercholesterolemi* OR hypercholesteremi* OR hypercholesterinaemi* OR hypercholesterinemi* OR hypercholesterolaemi* OR hyperlipidemi* OR hyperlipaemi* OR hyperlipoproteinemi* OR "hyperotosis corticalis generalisata familiaris" OR hypertriglyceridemi* OR hypertriglyceridaemi* OR hypolipaemi* OR hypolipidaemi* OR hypolipidemi* OR hypolipoproteinemi*or AND triglyceridemi* OR ("lipid metabolism" W/3 "inborn error*") OR ((lipemia OR lipaemia) W/1 familial) OR (("smith lemli opitz" OR acrodysgenital OR "rutledge friedman harrod" OR "rutledge lethal multiple congenital anomaly" OR "rsh slo" OR rsh OR slo) W/1 syndrome*) OR ("dehydrocholesterol reductase" W/1 deficienc*) OR hyperglycemi* OR hyperglycaemi* OR hyperglucemi* OR "hyper glycemi*" OR "elevated glucose blood" OR (insulin* W/2 (resistance* OR response* OR sensitiv*) OR hypertension* OR hypertensive* OR ((elevated OR high OR raised) W/2 "blood pressure*")))) AND (TITLE-ABS (((cancer* OR neoplasm* OR adenocarcinoma* OR adenoma* OR blastoma* OR carcinogens* OR carcinoid* OR carcinoma* OR carcinosarcoma* OR cyst OR cysts OR cystic OR malignant* OR malignanc* OR metastatic OR metastases OR metastasis OR neoplasia OR neoplastic* OR oncogene* OR oncogenic* OR precancer* OR "pre cancer*" OR praecancer* OR precarcinoma* OR "pre carcinoma*" OR praecarcinoma* OR premalignan* OR "pre malignan*" OR polycystic* OR pseudocyst* OR "pseudo cyst*" OR sarcoma* OR tumor OR tumors OR tumour OR tumours) W/5 ("alpha cell*" OR anal OR anus OR "beta cell*" OR breast* OR cervix OR cervical OR "circumanal gland*" OR "collecting duct*" OR colon* OR colorectal* OR "corpus luteum" OR diarrheogenic* OR ductal OR endometri* OR esophag* OR "gall bladder*" OR gallbladder* OR gastric* OR "gastrin produc*" OR "granulosa cell*" OR hepatic* OR hepatocellular* OR hypernephroid* OR "island cell*" OR "islet cell*" OR kidney OR liver* OR lobular* OR mammary* OR meninge* OR nephroid* OR oesophageal* OR ovarian* OR ovary* OR ovaries* OR pancrea* OR paratubal* OR parovari* OR "perianal gland*" OR rectal* OR rectum* OR renal OR sigmoid* OR stomach* OR "theca cell*" OR thyroid* OR uterine* OR uterus* OR "vasoactive intestinal peptide*" OR "vip secreting")) OR androblastoma* OR arrhenoblastoma* OR "cardia carcinoma*" OR "corpus uteri" OR cystosarcoma* OR "cysto sarcoma*" OR dysgerminoma* OR "gastric cardia" OR gastrinoma* OR "giant fibroadenoma" OR glucagonoma* OR hepatoma* OR hepatoblastoma* OR "hydatid* cyst* of morgagni*" OR "hydatid* of morgagni* hypernephroma*" OR insulinoma* OR insuloma* OR "kahler disease*" OR "leatherbottle stomach" OR "leather bottle stomach" OR (leukemia* W/2 (plasmacytic* OR "plasma cell*")) OR "linitis plastica" OR luteinoma* OR luteoma* OR "lynch* cancer*" OR meningioma* OR meningothelioma* OR "morbus kahler" OR "morgagni hydatid*" OR (myeloma* W/2 (multiple* OR multiplex* OR "plasma cell*")) OR "myeloma multiple*" OR myelomatos* OR nephroma* OR nephroblastoma* OR nesidioblastoma* OR "paget* disease*" OR "paget* nipple* disease*" OR (pancrea* W/2 (cholera* OR ipmn OR

ipmns)) OR (polypos* W/2 (adenomatous OR coli OR colon OR colus OR familial OR hereditary OR intestinal OR "myh associated")) OR "sclerocystic ovar*" OR somatostatinoma* OR thecoma* OR "thyroid nodule*" OR vipoma* OR "wagr comple*" OR ("watery diarrhea" W/3 (hypokalem* OR syndrome*)) OR (("denys-drash" OR drash OR gardner* OR grawitz* OR hboc OR lynch* OR meig* OR "Muir Torre" OR phyllodes OR phylloides OR "polycystic ovar*" OR "sertoli leydig" OR "stein leventhal" OR "Torre Muir" OR "verner morrison" OR wagr OR "wagr contiguous gene" OR wdha OR wilm*) W/2 (tumor* OR tumour* OR syndrome*))) OR ((adipose-related OR "adipose tissue related" OR fat-related OR "fat related" OR "obesity-related" OR "obesity related") W/6 (cancer* OR neoplasm*)) OR ((adipose-related OR "adipose tissue related" OR fat-related OR "fat related" OR "obesity-related" OR "obesity related") W/1 (adenocarcinoma* OR adenoma* OR blastoma* OR carcinogens* OR carcinoid* OR carcinoma* OR carcinosarcoma* OR cyst OR cysts OR cystic OR malignant* OR malignanc* OR metastatic OR metastases OR metastasis OR neoplasia OR neoplastic* OR oncogene* OR oncogenic* OR precancer* OR "pre cancer*" OR praecancer* OR precarcinoma* OR "pre carcinoma*" OR praecarcinoma* OR premalignan* OR "pre malignan*" OR polycystic* OR pseudocyst* OR "pseudo cyst*" OR sarcoma* OR tumor OR tumors OR tumour OR tumours))) AND (TITLE-ABS ("systematic review*" OR "data synthes*" OR "data extraction*" OR "data abstraction*" OR handsearch* OR "hand search*" OR "mantel haenszel" OR peto OR "der simonian" OR dersimonian OR "fixed effect*" OR "latin square*" OR "met analy*" OR metanaly* OR "meta regression*" OR metaregression* OR "meta-analy*" OR metaanaly*) OR TITLE-ABS (((systematic*) W/3 (review* OR overview* OR umbrella*)) OR ((methodologic*) W/3 (review* OR overview* OR umbrella*)) OR ((review*) W/2 (umbrella* OR overview*)) OR ((quantitative) W/3 (review* OR overview* OR synthes*)) OR ((research) W/3 (integrati* OR overview*)) OR ((integrative) W/3 (review* OR overview*)) OR ((collaborative) W/3 (review* OR overview*)) OR ((pool*) W/3 (analy*)))))

Initial Results: 97 documents on February 10, 2021

Updated Results: 30 documents on January 3, 2023, with (LIMIT-TO (PUBYEAR , 2022) OR LIMIT-TO (PUBYEAR , 2021))

Results: 127 documents

Scopus Legend

Field codes: TITLE-ABS-KEY = Document Title, Abstract, Keywords

Proximity operator: W/

Truncation: *

Appendix C: Bibliography of Included Systematic Reviews with Meta-analysis

1. Bhandari R, Kelley GA, Hartley TA, Rockett IR. Metabolic syndrome is associated with increased breast cancer risk: a systematic review with meta-analysis. *Int J Breast Cancer*. 2014;2014:189384. doi:10.1155/2014/189384.

2. Chen Y, Li X, Wu S, Ye W, Lou L. Metabolic syndrome and the incidence of hepatocellular carcinoma: a meta-analysis of cohort studies. *Onco Targets Ther*. 2018;11:6277-6285. doi:10.2147/OTT.S154848.

3. Du W, Guo K, Jin H, Sun L, Ruan S, Song Q. Association Between Metabolic Syndrome and Risk of Renal Cell Cancer: A Meta-Analysis. *Front Oncol*. 2022;12:928619. doi:10.3389/fonc.2022.928619.

4. Esposito K, Chiodini P, Capuano A, Bellastella G, Maiorino MI, Giugliano D. Metabolic syndrome and endometrial cancer: a meta-analysis. *Endocrine*. 2014;45(1):28-36. doi:10.1007/s12020-013-9973-3.

5. Esposito K, Chiodini P, Capuano A, Bellastella G, Maiorino MI, Rafaniello C, Giugliano D. Metabolic syndrome and postmenopausal breast cancer: systematic review and meta-analysis. *Menopause*. 2013;20(12):1301-9. doi:10.1097/GME.0b013e31828ce95d.

6. Esposito K, Chiodini P, Capuano A, et al. Colorectal cancer association with metabolic syndrome and its components: a systematic review with meta-analysis. *Endocrine*. 2013;44(3):634-47. doi:10.1007/s12020-013-9939-5.

7. Esposito K, Chiodini P, Colao A, Lenzi A, Giugliano D. Metabolic syndrome and risk of cancer: a systematic review and meta-analysis. *Diabetes Care*. 2012;35(11):2402-11. doi:10.2337/dc12-0336.

8. Guo M, Liu T, Li P, et al. Association Between Metabolic Syndrome and Breast Cancer Risk: An Updated Meta-Analysis of Follow-Up Studies. *Front Oncol*. 2019;9:1290. doi:10.3389/fonc.2019.01290.

9. Han F, Wu G, Zhang S, Zhang J, Zhao Y, Xu J. The association of Metabolic Syndrome and its Components with the Incidence and Survival of Colorectal Cancer: A Systematic Review and Meta-analysis. *International Journal of Biological Sciences*. 2021;17(2):487-497. doi:10.7150/ijbs.52452.

10. Jinjuvadia R, Lohia P, Jinjuvadia C, Montoya S, Liangpunsakul S. The association between metabolic syndrome and colorectal neoplasm: systemic review and meta-analysis. *J Clin Gastroenterol*. 2013;47(1):33-44. doi:10.1097/MCG.0b013e3182688c15.

11. Jinjuvadia R, Patel S, Liangpunsakul S. The association between metabolic syndrome and hepatocellular carcinoma: systemic review and meta-analysis. *J Clin Gastroenterol*. 2014;48(2):172-7. doi:10.1097/MCG.0b013e3182a030c4.

12. Li Y, Shi J, Liu X, Deng Q, Huang Y, Yang Z. Metabolic syndrome relates to high risk in hepatocellular carcinoma: a meta-analysis. *Discov Med*. 2018;26(144):185-196.

13. Lu B, Qian JM, Li JN. The metabolic syndrome and its components as prognostic factors in colorectal cancer: A meta-analysis and systematic review. *J Gastroenterol Hepatol*. 2022;26:26. doi:10.1111/jgh.16042.

14. Lu L, Koo S, McPherson S, Hull MA, Rees CJ, Sharp L. Systematic review and meta-analysis: Associations between metabolic syndrome and colorectal neoplasia outcomes. *Colorectal Dis*. 2022;24(6):681-694. doi:10.1111/codi.16092.

15. Ren H, Wang J, Gao Y, Yang F, Huang W. Metabolic syndrome and liver-related events: a systematic review and meta-analysis. *BMC Endocr Disord*. 2019;19(1):40. doi:10.1186/s12902-019-0366-3.

16. Shen X, Wang Y, Zhao R, Wan Q, Wu Y, Zhao L, Wu X. Metabolic syndrome and the risk of colorectal cancer: a systematic review and meta-analysis. *Int J Colorectal Dis*. 2021;36(10):2215-2225. doi:10.1007/s00384-021-03974-y.

17. Tao W, Yuan C, Kang B, et al. The Effect of Metabolic Syndrome on Colorectal Cancer Prognosis after Primary Surgery. *Nutr Cancer*. 2023;75(1):331-338. doi:10.1080/01635581.2022.2112243.

18. Wang L, Du ZH, Qiao JM, Gao S. Association between metabolic syndrome and endometrial cancer risk: a systematic review and meta-analysis of observational studies. *Aging*. 2020;12(10):9825-9839. doi:10.18632/aging.103247.

19. Yin DT, He H, Yu K, et al. The association between thyroid cancer and insulin resistance, metabolic syndrome and its components: A systematic review and meta-analysis. *International Journal Of Surgery*. 2018;57:66-75. doi:10.1016/j.ijsu.2018.07.013.

20. Zhang J, Wu H, Wang R. Metabolic syndrome and esophageal cancer risk: a systematic review and meta-analysis. *Diabetol Metab Syndr*. 2021;13(1):8. doi:10.1186/s13098-021-00627-6.

21. Zhao P, Xia N, Zhang H, Deng T. The Metabolic Syndrome Is a Risk Factor for Breast Cancer: A Systematic Review and Meta-Analysis. *Obesity Facts*. 2020;13(4):384-396. doi:10.1159/000507554.

Appendix D: Bibliography of excluded publications at the full-text review stage with reasons for exclusion.

1. Adambekov S, Yi Y, Fabio A, Miljkovic I, Edwards RP, Lopa S, Linkov F. Metabolic Syndrome in Endometrial Cancer Patients: Systematic Review. *Metab Syndr Relat Disord*. 2019;17(5):241-249. doi:10.1089/met.2018.0106.
Exclusion reason: Wrong study design.

2. Aleksandrova K, Nimptsch K, Pischon T. Influence of Obesity and Related Metabolic Alterations on Colorectal Cancer Risk. *Current Nutrition Reports*. 2013;2(1):1-9. doi:10.1007/s13668-012-0036-9.
Exclusion reason: Wrong study design.

3. Eskandari D, Khodabandehloo N, Gholami A, Samadanifard H, Hejrati A. Investigation of the association between metabolic syndrome and breast cancer patients. *Eur J Transl Myol*. 2020;30(1):8776. doi:10.4081/ejtm.2019.8776.
Exclusion reason: Wrong study design.

4. Esposito K, Capuano A, Giugliano D. Metabolic syndrome and cancer: Holistic or reductionist? *Endocrine*. 2014;45(3):362-364. doi:10.1007/s12020-013-0056-2.
Exclusion reason: Wrong study design.

5. Esposito K, Giugliano D. The association between metabolic syndrome and hepatocellular carcinoma: a missed meta-analysis. *J Clin Gastroenterol*. 2014;48(8):742-3. doi:10.1097/MCG.0000000000000097.
Exclusion reason: Wrong study design.

6. Hu D, Zhang M, Zhang H, et al. Prediction of Metabolic Syndrome for the Survival of Patients With Digestive Tract Cancer: A Meta-Analysis. *Front Oncol*. 2019;9:281. doi:10.3389/fonc.2019.00281.
Exclusion reason: Wrong study design.

7. Jung HS, Myung SK, Kim BS, Seo HG. Metabolic syndrome in adult cancer survivors: a meta-analysis. *Diabetes Res Clin Pract*. 2012;95(2):275-82. doi:10.1016/j.diabres.2011.08.029.
Exclusion reason: Wrong outcomes.

8. Li P, Wang T, Zeng C, Yang M, Li G, Han J, Wu W. Association between metabolic syndrome and prognosis of breast cancer: a meta-analysis of follow-up studies. *Diabetol Metab Syndr*. 2020;12:10. doi:10.1186/s13098-019-0514-y.
Exclusion reason: Wrong exposures.

9. Li Z, Han H, Chang Y. Association between metabolic syndrome and the incidence of gastric cancer: a meta-analysis of cohort studies. *Diabetol Metab Syndr*. 2019;11:83. doi:10.1186/s13098-019-0478-y.
Exclusion reason: Wrong outcomes.

10. Lindkvist B, Almquist M, Bjorge T, et al. Prospective cohort study of metabolic risk factors and gastric adenocarcinoma risk in the Metabolic Syndrome and Cancer Project (Me-Can). *Cancer Causes Control*. 2013;24(1):107-16. doi:10.1007/s10552-012-0096-6.
Exclusion reason: Wrong intervention.

11. Lubián López DM, Castillo Lara M, Rodríguez Rodríguez B, Butrón Hinojo CA, Martínez Herrera A, Sánchez Borrego R, Mendoza Ladrón de Guevara N. Metabolic syndrome and prognostic factors in postmenopausal breast cancer patients. *Breast Journal*. 2019;25(3):548-551. doi:10.1111/tbj.13280.
Exclusion reason: Wrong study design.

12. Luo Y, Liu JS, Dai B, Qian K. The influence of metabolic syndrome on gastric cancer: A meta-analysis. *Asian J Surg*. 2021;44(12):1596-1597. doi:10.1016/j.asjsur.2021.08.014.
Exclusion reason: Wrong outcomes.

13. Mariani M, Sassano M, Boccia S. Metabolic syndrome and gastric cancer risk: a systematic review and meta-analysis. *Eur J Cancer Prev*. 2020;16:16. doi:10.1097/CEJ.0000000000000618.
Exclusion reason: Wrong outcomes.

14. Mili N, Paschou SA, Goulis DG, Dimopoulos MA, Lambrinoudaki I, Psaltopoulou T. Obesity, metabolic syndrome, and cancer: pathophysiological and therapeutic associations. *Endocrine*. 2021;74(3):478-497. doi:10.1007/s12020-021-02884-x.
Exclusion reason: Wrong study design.

15. O'Sullivan DE, Sutherland RL, Town S, et al. Risk factors for early-onset colorectal cancer: A systematic review and meta-analysis. *Clin Gastroenterol Hepatol*. 2021;29:29. doi:10.1016/j.cgh.2021.01.037.
Exclusion reason: Wrong study design.

16. Rosato V, Tavani A, Bosetti C, et al. Metabolic syndrome and pancreatic cancer risk: a case-control study in Italy and meta-analysis. *Metabolism*. 2011;60(10):1372-8. doi:10.1016/j.metabol.2011.03.005.
Exclusion reason: Wrong study design.

17. Srinivasan M, Arzoun H, Gk LB, Thangaraj SR. A Systematic Review: Does Insulin Resistance Affect the Risk and Survival Outcome of Breast Cancer in Women? *Cureus*. 2022;14(1):e21712. doi:10.7759/cureus.21712.
Exclusion reason: Wrong study design.

18. Stocks T, Bjorge T, Ulmer H, et al. Metabolic risk score and cancer risk: pooled analysis of seven cohorts. *Int J Epidemiol*. 2015;44(4):1353-63. doi:10.1093/ije/dyv001.
Exclusion reason: Wrong intervention.

19. Uzunlulu M, Telci Caklili O, Oguz A. Association between Metabolic Syndrome and Cancer. *Ann Nutr Metab*. 2016;68(3):173-179. doi:10.1159/000443743.

Exclusion reason: Wrong study design.

20. Wu H, Zhang J, Zhou B. Metabolic syndrome and colorectal adenoma risk: A systematic review and meta-analysis. *Clinics & Research in Hepatology & Gastroenterology*. 2021;45(5):101749. doi:10.1016/j.clinre.2021.101749.

Exclusion reason: Wrong outcomes.

Table S1: Quality assessment of studies included in the umbrella review using the A MeaSurement Tool to Assess systematic Reviews (AMSTAR) 2 criteria.

Source	Registered protocol prior to conducting the review	Adequate Literature Search	Justification for excluded studies	Risk of bias from individual studies being included in the review	Appropriateness of meta-analytical methods	Potential impact of risk of bias on the results of the meta-analysis	Assessment of presence and likely impact of publication bias	Overall AMSTAR 2 Rating
Bhandari et al, 2014	*No*	*No*	*Yes*	*Yes*	*Yes*	*No*	*Yes*	*Critically low*
Chen et al, 2018	*No*	*Yes*	*No*	*Yes*	*Yes*	*Yes*	*Yes*	*Critically low*
Duet al, 2022	*No*	*Partial Yes*	*No*	*Yes*	*Yes*	*Yes*	*Yes*	*Critically low*
Esposito et al, 2012	*No*	*Yes*	*No*	*No*	*Yes*	*Yes*	*Yes*	*Critically low*
Esposito et al, 2013 (CRC)	*No*	*Partial Yes*	*No*	*Yes*	*Yes*	*Yes*	*Yes*	*Critically low*
Esposito et al, 2013 (Breast)	*No*	*Partial Yes*	*No*	*Yes*	*Yes*	*Yes*	*Yes*	*Critically low*
Esposito et al, 2014	*No*	*Partial Yes*	*No*	*Yes*	*Yes*	*Yes*	*Yes*	*Critically low*
Guo et al, 2019	*No*	*No*	*No*	*Yes*	*Yes*	*Yes*	*Yes*	*Critically low*
Han et al, 2021	*No*	*Yes*	*No*	*Yes*	*Yes*	*Yes*	*Yes*	*Critically low*
Jinjuvadia et al, 2013	*No*	*Yes*	*No*	*Yes*	*Yes*	*Yes*	*Yes*	*Critically low*
Jinjuvadia et al, 2014	*No*	*Partial Yes*	*No*	*Yes*	*Yes*	*Yes*	*Yes*	*Critically low*
Li et al, 2018	*No*	*Yes*	*No*	*Yes*	*Yes*	*Yes*	*No*	*Critically low*
Lu B et al, 2022	*Yes*	*Partial Yes*	*No*	*Yes*	*Yes*	*Yes*	*Yes*	*Critically low*
Lu L et al, 2022	*Yes*	*No*	*No*	*Yes*	*Yes*	*Yes*	*No*	*Critically low*
Ren et al, 2019	*No*	*Partial Yes*	*No*	*Yes*	*Yes*	*Yes*	*Yes*	*Critically low*
Shen et al, 2021	*No*	*Yes*	*No*	*Yes*	*Yes*	*Yes*	*Yes*	*Critically low*
Tao et al, 2023	*No*	*No*	*No*	*Yes*	*Yes*	*Yes*	*No*	*Critically low*
Wang et al, 2020	*No*	*Yes*	*No*	*Yes*	*Yes*	*Yes*	*No*	*Critically low*
Yin et al, 2018	*No*	*No*	*No*	*Yes*	*Yes*	*No*	*Yes*	*Critically low*
Zhang et al, 2021	*No*	*No*	*No*	*Yes*	*Yes*	*Yes*	*Yes*	*Critically low*
Zhao et al, 2020	*No*	*No*	*No*	*Yes*	*Yes*	*Yes*	*Yes*	*Critically low*

Table S2: Quality assessment of studies included in the umbrella review using the A MeaSurement Tool to Assess systematic Reviews (AMSTAR) 2 criteria, removing two critical domains[a].

Article	Adequate Literature Search	Risk of bias from individual studies being included in the review	Appropriateness of meta-analytical methods	Potential impact of risk of bias on the results of the meta-analysis	Assessment of presence and likely impact of publication bias	Overall AMSTAR2 Rating
Bhandari 2014	*No*	*Yes*	*Yes*	*No*	*Yes*	*Critically low*
Hu 2019	*No*	*No*	*Yes*	*Yes*	*Yes*	*Critically low*
Lu L 2022	*No*	*Yes*	*Yes*	*Yes*	*No*	*Critically low*
Rosato 2011	*Yes*	*No*	*Yes*	*Yes*	*No*	*Critically low*
Tao 2023	*No*	*Yes*	*Yes*	*Yes*	*No*	*Critically low*
Yin 2018	*No*	*Yes*	*Yes*	*No*	*Yes*	*Critically low*
Esposito 2012	*Yes*	*No*	*Yes*	*Yes*	*Yes*	*Low*
Guo 2019	*No*	*Yes*	*Yes*	*Yes*	*Yes*	*Low*
Li 2018	*Yes*	*Yes*	*Yes*	*Yes*	*No*	*Low*
Wang 2020	*Yes*	*Yes*	*Yes*	*Yes*	*No*	*Low*
Zhang 2021	*No*	*Yes*	*Yes*	*Yes*	*Yes*	*Low*
Zhao 2020	*No*	*Yes*	*Yes*	*Yes*	*Yes*	*Low*
Du2022	*Partial Yes*	*Yes*	*Yes*	*Yes*	*Yes*	*Moderate*
Esposito 2013 (CRC)	*Partial Yes*	*Yes*	*Yes*	*Yes*	*Yes*	*Moderate*
Esposito 2013 (Breast)	*Partial Yes*	*Yes*	*Yes*	*Yes*	*Yes*	*Moderate*
Esposito 2014	*Partial Yes*	*Yes*	*Yes*	*Yes*	*Yes*	*Moderate*
Jinjuvadia 2014	*Partial Yes*	*Yes*	*Yes*	*Yes*	*Yes*	*Moderate*
Lu B 2022	*Partial Yes*	*Yes*	*Yes*	*Yes*	*Yes*	*Moderate*
Ren 2019	*Partial Yes*	*Yes*	*Yes*	*Yes*	*Yes*	*Moderate*
Chen 2018	*Yes*	*Yes*	*Yes*	*Yes*	*Yes*	*High*
Han 2021	*Yes*	*Yes*	*Yes*	*Yes*	*Yes*	*High*
Jinjuvadia 2013	*Yes*	*Yes*	*Yes*	*Yes*	*Yes*	*High*
Shen 2021	*Yes*	*Yes*	*Yes*	*Yes*	*Yes*	*High*

[a]Removing two critical domains, including 1) Registered protocol prior to conducting the review, and 2) Provided justification for excluded studies.

Table S3: Strength and certainty of evidence including only cohort studies evaluating metabolic syndrome with obesity-related cancer risk.

Study design	ORC (risk)	Author, Year	N studies	HR	95% CI	p-value	PI	Total N	Cases	I2	Egger's	ESB	Largest CI	Strength of evidence
Cohort	Breast	Guo et al. 2019	8	1.14	1.05-1.25	2.30E-03	0.93-1.40	505041	9386	72%	0.0542	0.1893	0.97-1.12	IV
All	Breast	Guo et al. 2019	17	1.27	1.12-1.43	1.4E-04	0.87-1.85	507146	10058	75%	7.20E-03	8.0E-06	0.97-1.12	III
Cohort	CRC	Shen et al. 2021	10	1.28	1.19-1.38	1.21E-10	1.04-1.58	26911701	167413	72%	1.78E-01	2.90E-01	1.19-1.23	II
All	CRC	Shen et al. 2021	21	1.41	1.31-1.52	3.0E-19	1.04-1.91	27078880	186123	77%	1.70E-03	5.33E-02	1.19-1.23	II
Cohort	EAC	Zhang et al. 2021	3	1.45	1.16-1.82	1.27E-03	0.34-6.29	770554	219	0%	1.38E-01	6.16E-01	1.19-2.05	IV
All	EAC	Zhang et al. 2021	5	1.21	1.02-1.43	2.75E-02	0.76-1.92	793049	3978	31%	5.36E-01	2.9E-01	1.19-2.05	IV
Cohort	Endometrial	Wang et al. 2020	2	1.96	1.37-2.81	2.22E-04	NA	29738	196	42%	NA	NA	0.98-2.49	IV
All	Endometrial	Wang et al. 2020	6	1.49	1.23-1.80	5.4E-05	0.81-2.74	149895	17768	79%	2.64E-02	8.1E-02	1.12-1.24	III
Cohort	Pancreatic	Esposito et al. 2012	3	1.31	1.16-1.48	1.08E-05	0.60-2.89	323829	605	28%	8.33E-01	9.10E-02	1.14-1.48	IV
All	Pancreatic	Esposito et al. 2012	4	1.33	1.18-1.49	3.3E-06	1.02-1.72	324177	779	54%	9.94E-01	3.5E-01	1.14-1.48	IV
Cohort	Thyroid	Yin et al. 2018	1	1.06	0.93-1.22	3.89E-01	NA	289866	133	NA	NA	NA	NA	ns
All	Thyroid	Yin et al. 2018	2	1.07	0.94-1.22	3.3E-01	N/A	289948	174	0%	N/A	6.4E-01	0.93-1.22	ns
Cohort	Renal	Du et al. 2022	7	1.62	1.31-2.01	9.45E-06	0.86-3.05	6231464	10192	86%	7.15E-01	1.28E-01	1.26-1.45	III
All	Renal	Du et al. 2022	8	1.67	1.40-2.00	1.9E-08	0.98-2.85	6644139	17025	86%	2.39E-01	2.2E-01	1.26-1.45	II

Abbreviations: ORC, obesity-related cancer; HR, hazard ratio; CI, confidence interval; PI, prediction interval; ESB, excess significance.
